# Effect of Oregon grape root extracts on P-glycoprotein mediated transport in *in vitro* cell lines

**DOI:** 10.3389/jpps.2023.11927

**Published:** 2024-01-18

**Authors:** Ying Fan, Zhu Zhou, Lei Zhang

**Affiliations:** ^1^ Division of Clinical Review, Office of Safety and Clinical Evaluation, Office of Generic Drugs, Center for Drug Evaluation and Research, U.S. Food and Drug Administration, Silver Spring, MD, United States; ^2^ York College, The City University of New York, Jamaica, NY, United States; ^3^ Office of Research and Standards, Office of Generic Drugs, Center for Drug Evaluation and Research, U.S. Food and Drug Administration, Silver Spring, MD, United States

**Keywords:** Oregon grape root, berberine, berbamine, P-glycoprotein (P-gp), cyclosporin A (CsA), digoxin, inhibition, induction

## Abstract

**Purpose:** This study aims to investigate the potential of Oregon grape root extracts to modulate the activity of P-glycoprotein.

**Methods:** We performed ^3^H-CsA or ^3^H-digoxin transport experiments in the absence or presence of two sources of Oregon grape root extracts (E1 and E2), berberine or berbamine in Caco-2 and MDCKII-MDR1 cells. In addition, real time quantitative polymerase chain reaction (RT-PCR) was performed in Caco-2 and LS-180 cells to investigate the mechanism of modulating P-glycoprotein.

**Results:** Our results showed that in Caco-2 cells, Oregon grape root extracts (E1 and E2) (0.1–1 mg/mL) inhibited the efflux of CsA and digoxin in a dose-dependent manner. However, 0.05 mg/mL E1 significantly increased the absorption of digoxin. Ten µM berberine and 30 µM berbamine significantly reduced the efflux of CsA, while no measurable effect of berberine was observed with digoxin. In the MDCKII-MDR1 cells, 10 µM berberine and 30 µM berbamine inhibited the efflux of CsA and digoxin. Lastly, in real time RT-PCR study, Oregon grape root extract (0.1 mg/mL) up-regulated mRNA levels of human *MDR1* in Caco-2 and LS-180 cells at 24 h.

**Conclusion:** Our study showed that Oregon grape root extracts modulated P-glycoprotein, thereby may affect the bioavailability of drugs that are substrates of P-glycoprotein.

## Introduction

Oregon grape root (*Mahonia aquifolia*), has synergistic antibacterial, anti-inflammatory, and bile-stimulating properties and is used for chronic eruptions, rashes associated with pustules, and rashes associated with eating fatty foods [1]. Recently, Oregon grape root extract has been shown to effectively treat inflammatory skin diseases, such as psoriasis and eczema [2, 3]. The Oregon grape root extracts are available in both oral and topical dosage forms.

Berberine, a bisbenzylisoquinoline alkaloid, is found in Oregon grape root and has been used for many years in traditional Eastern medicine as an effective treatment of gastroenteritis and diarrhea [4]. Since then, other pharmacological effects have been reported such as antimicrobial [5], antiarrhythmic [6], anticancer [7], anti-inflammatory [8] and antiproliferative effects [9]. Studies have shown that berberine is a weak to moderate inhibitor of CYP1As, CYP2C8, and CYP2E1 [10]. Additionally, berberine not only exhibited time-dependent inhibition of CYP2D6 and CYP3A4 but also showed strong inactivation of CYP2D6 and modest inactivation of CYP3A [10, 11]. Berbamine, another bisbenzylisoquinoline alkaloid, although was reported to be present in Oregon grape root, is not a typical component of Oregon grape root. It is found in other plant species such as Amur barberry (*Berberis amurensis*). Berbamine is widely used in traditional Chinese medicine as an antiarrhythmic, antihypertensive, anti-inflammatory and anticancer agent [12, 13]. Particularly, berbamine showed antiproliferative effects for melanoma, chronic myeloid leukemia and breast cancer [13]. Previous phenotyping studies indicate that CYP3A4 is the primary enzyme involved in the metabolism of berbamine [13].

*In vivo* experiments using P-glycoprotein (P-gp)-knock out mice and *in vitro* studies using both Caco-2 cells and MDR1-transfected MDCK cells suggested berberine is a P-gp substrate [14–17]. P-gp is a member of the ATP-binding cassette (ABC) transporter family, also known as ABCB1 or MDR1 (encoded by *ABCB1* or *MDR1* gene). In Caco-2 cells, P-gp inhibitors such as verapamil (1 mM), daunomycin (1 mM) and rhodamine123 (1 mM) were able to inhibit the efflux of 20 µM berberine by 100% [18]. Similarly, in the presence of the P-gp inhibitors cyclosporin A (10 µM), the efflux ratios of berberine (10 µM) were significantly reduced to 0.79 and 1.34, respectively [17]. In addition to P-gp, previous studies showed that human organic cation transporter 2 (OCT2, SLC22A2), OCT3 (SLC22A3) and multidrug and toxin extrusion protein 1 (MATE1) mediated the transport of berberine in the kidney [19–22], and OCT1, MATE1, organic anion-transporting polypeptide 1B3 (OATP1B3) mediated transport of berberine in the liver [23, 24]. Meanwhile, berberine is not an OATP1B1 and multidrug resistance-associated protein 2 (MRP2) substrate [17, 23]. Conversely, no significant transport of berberine was observed by bile salt export pump (BSEP), MRP2, MRP3, and sodium/taurocholate co-transporting polypeptide (NTCP) *in vitro* [25–27].

As an inhibitor for transporters, berberine inhibits breast cancer resistance protein (BCRP), MATE1, MATE2-K, organic anion transporter 1 (OAT1), OAT3, OCT1, OCT2, and OCT3 *In vitro* [19, 27–30]. There are inconsistent data about whether berberine can inhibit P-gp, OATP1B1, OTAP1B3, or OATP2B1 [17, 26, 27, 31]. Studies have shown that berberine can induce P-gp. Twenty-four hours exposure of berberine up-regulated P-gp expression in human and murine hepatoma cells [32] as well as up-regulated P-gp expression in cultured bovine brain capillary endothelial cells [33]. Jing *et al.* indicated that P-gp protein was upregulated by berberine treatment (0.1, 0.5 or 2.5 µM up to 48 h) in a dose- and time-dependent manner in Caco-2 cells [34]. Seven day pretreatment of 30 µM berberine increased the efflux of daunomycin in Caco-2 cells by 1.4 fold [18].

Berbamine has, on the other hand, been shown to down-regulate the expression of MDR1 mRNA after 72 h treatment in human erythroleukemic cells [35] as well as modulating multi-drug resistance (MDR) in breast cancer cells [36]*. In vitro* studies with berbamine have shown an increase in the intracellular concentration of adriamycin and down-regulation of MDR1 mRNA and P-gp levels in a human leukemic cell line, K562/A02 [35] as well as in a human breast cancer cell line, MCF7 [37].

To our best knowledge, there are no studies examine the effects of Oregon grape root extracts on P-gp. Because Oregon grape root extract is known as a source of berberine and not berbamine, we hypothesized that Oregon grape root extracts (e.g., E1 and E2) will have similar effects as berberine in the modulation of P-gp (inhibition or upregulation). Herein, we have conducted studies: i) to confirm the presence of berberine and but not beramine in Oregon grape root extracts; ii) to evaluate the effect of the Oregon grape root extracts on the transport of two well known P-gp substrates, CsA and digoxin, using the Caco-2 drug transport models; and iii) to evaluate the effect of the Oregon grape root extracts on the gene expression of human *MDR1* in Caco-2 and LS-180 cells to elucidate the role of PXR in the mechanism of P-gp induction. Additionally, we included berberine and berbamine in our *in vitro* studies to confirm their interactions with P-gp as a substrate and inhibitor in Caco-2 and MDCK II-MDR1 cells.

## Materials and methods

### Chemicals

Radiolabeled ^3^H-cyclosporin A (CsA, 7.0 Ci/mmol, 96.7% purity) and ^3^H-digoxin (23.5 Ci/mmol, 97% purity) were obtained from Amersham, Inc. (Piscataway, NJ, USA). Berberine chloride, berbamine dihydrochloride, CsA, digoxin, verapamil, levothyroxine, rifampin, sodium pyruvate, L-lactic dehydrogenase (LDH), β-nicotinamide adenine dinucleotide, reduced form (β-NADH), thiazolyl blue tetrazolium bromide and triethylamine (TEA) were purchased from Sigma, Inc. (St. Louis, MO, USA). Fetal bovine serum (FBS) was purchased from Hyclone (Logan, UT, USA). Dulbecco’s Modified Eagle Medium (DMEM) and non-essential amino acids were obtained from Invitrogen Corporation (Grand Island, NY, USA). Penicillin/streptomycin solution was purchased from Mediatech, Inc. (Herndone, VA, USA). The 18s internal standard and *MDR1* primers were purchased from Applied Biosystems (Foster City, CA, USA). Solvent acetonitrile was HPLC grade and was purchased from Fisher Scientific (Fair Lawn, NJ, USA). Ammonium acetate was purchased from Mallinckroft Baker Inc. (Paris, KY, USA). Oregon grape root extract 1 was a gift from Oregon’s Wild Harvest (Sandy, OR, USA). Oregon grape root extract 2 was purchased as a root powder from Health Herbs (Philomath, OR, USA). All other reagents were analytical grade.

### Oregon grape root extract preparation for the experiments

See the details on the preparation of Oregon grape root extract 1 (E1) (liquid) and Oregon grape root extract 2 (E2) (root powder) for the experiments in the Supplementary Material.

### Cell culture

The human intestinal epithelial cell line, LS-180 was purchased from American Type Culture Collection (ATCC) (Manassas, VA, USA) and grown in Minimum Essential Medium supplemented with 10% FBS. The Caco-2 cells were obtained from ATCC. MDCKII-MDR1 and MDCKII wild-type cell lines were a generous gift from Dr. Piet Borst (The Netherlands Cancer Research Institute). Both Caro-2 and MDCKII cells were grown in DMEM, 10% FBS, 0.1 mM non-essential amino acids, and 0.01% penicillin/streptomycin [38,39]. All cells were maintained at 37°C with 5% CO_2_, and 95% relative humidity. For the transport experiments, cells were seeded, 3.0 × 10^5^cells/well, onto 6-well Transwell^®^ inserts 4.71 cm^2^ (Corning Costar, Cambridge, MA, USA) and maintained until use on Days 21–25 (Caco-2, passages 23–30) and Days 6–7 (MDCKII-MDR1 and MDCKII wild-type). The integrity of the monolayers was assessed by measuring transepithelial electrical resistance (TEER) using a World Precision Instrument, EVOM (Sarasota, FL, USA) and evaluating ^14^C-mannitol transport. The average TEER readings minus the background were 625.94 ± 75.47 Ωcm^2^ (Caco-2), 476.91 ± 101.21 Ωcm^2^ (MDCKII-MDR1) and 211.52 ± 22.33 Ωcm^2^ (MDCKII wild-type). Also, permeability studies with ^14^C-mannitol were performed with the various treatment conditions and the transport rate was less than 1% per hour throughout the entire experiment. The apparent permeability (P_app_) of mannitol was 2.09 ± 0.40 × 10^-6^ cm/s, 2.38 ± 0.13 × 10^−6^ cm/s, and 2.16 ± 0.72 × 10^−6^ cm/s for Caco-2, MDCKII-MDR1, and MDCKII wild-type cells, respectively. These data indicate that the cell monolayer was not compromised. For the real time RT-PCR, cells were seeded at 6 × 10^5^ cells/well (Caco-2) and 1 × 10^6^ cells/well (LS-180) onto 6-well Transwell^®^ inserts 4.71 cm^2^ and grown for 7 (Caco-2) and 6 (LS-180) days.

### Chemical exposure

We initiated cytotoxicity experiments with the Caco-2, MDCKII-MDR1 and MDCKII-wild type cells on Day 7 (Caco-2) and Day 4 (MDCKII-MDR1 and MDCKII-wild type) after the initial plating of the cells at 2.5 × 10^3^ cells/well in 48-well plates (Becton Dickinson, Franklin Lakes, NJ, USA). See details of preparation for the cytotoxicity experiments in Supplementary Material.

For the transport experiments, cells grown on Transwell^®^ inserts were exposed to test compounds on 21–25 days (Caco-2) or 6–7 days (MDCKII-MDR1 and MDCKII-wild type). Stock solutions of 10 mM berberine or berbamine were prepared as described above. Stock solutions of 100 mg/mL E1 or E2 were prepared by dissolving the 100 mg E1 or E2 in 25% ethanol. The stock solutions of berberine, berbamine, E1 and E2 were further diluted with HBSS at a pH of 6.8 for the apical (A) compartment and 7.4 for the basolateral (B) compartment to yield final concentrations of 3, 10, 30, and 100 µM berberine and berbamine and 0.05, 0.1, 0.25, 0.5 and 1 mg/mL for E1 and E2. Ethanol (0.15%v/v) was used as the solvent vehicle for CsA and verapamil and DMSO (0.15%v/v) were used for digoxin and berberine. The MDR1 transport inhibitor, 100 µM verapamil, was used as a positive control and no treatment was used as a negative control.

For the real time RT-PCR studies, 6 × 10^5^ cells/well (Caco-2) or 1 × 10^6^ cells/well (LS-180) were grown on Transwell^®^ inserts and exposed to test compounds on Day 7 (Caco-2) or Day 6 (LS-180). Stock solutions of 10 mM levothyroxine or 10 µM rifampin were prepared by dissolving compounds in 50% DMSO with 50% distilled water. The stock solutions of E1, E2, levothyroxine or rifampin were further diluted with serum-free and antibiotic-free medium to yield final concentrations of 0.1 mg/mL and 0.25 mg/mL for E1 and E2 and 100 µM levothyroxine or 10 µM rifampin. The final treatment concentration of ethanol or DMSO was 0.15% (v/v) ethanol or 0.5% (v/v) DMSO/culture medium. Levothyroxine and rifampin were used as positive controls for Caco-2 and LS-180 cells, respectively.

### Cytotoxicity assays

#### Lactate dehydrogenase (LDH) assay and MTT assay

See details of LDH assay and MTT assay methods in the Supplementary Material.

### Transport studies

Transport of ^3^H-CsA and ^3^H-digoxin (0.5 µM and 1.0 µCi), MDR1 substrates, were performed using transport buffer consisting of HBSS with 10 mM HEPES and 25 mM D-glucose at a pH of 6.8 for the apical (A) compartment and 7.4 for the basolateral (B) compartment. Experiments were performed at 37°C, 5% CO_2_, and 95% relative humidity. Cells in the Transwell^®^ inserts were washed 3 times with a transport buffer and allowed to equilibrate for 30 min prior to the addition of test compounds. Berberine, berbamine, E1, E2 and verapamil were prepared in a transport buffer at the appropriate pH (pH 6.8 for the A compartment and 7.4 for the B compartment) and allowed to incubate at 37°C for 30 min prior to the start of the experiment. Berberine, berbamine, E1, E2 and verapamil were present in both the A and B chambers during the transport of the MDR1 substrates. To evaluate the efflux mechanism involved with berberine and berbamine, 100 µM of berberine and berbamine were placed in the donor chamber and 100 µL samples were collected in the receiver chamber at 0, 0.5, 1.0, 1.5, 2.0, 2.5, and 3.0 h and analyzed by HPLC. Each experiment was performed in duplicate and repeated for each condition tested. For experiments performed in “non-sink” conditions, 100 µL aliquots were taken from the donor and receiver chambers at the beginning and from the receiver chamber at 0.5, 1.0, 1.5, 2.0, 2.5, and 3.0 h. An equal volume of 100 µL was replaced in the chamber at each sample time point. “Non-sink” conditions were performed when less than 10% of compound is transported across cells during the transport experiment such that a concentration gradient was always present. For the “sink” condition experiments, the entire receiver volume was replaced at each time interval and a 100 µL aliquot was taken from the receiver volume for scintillation counting. A 100 µL sample was taken from the donor chamber at 3 h in order to calculate the mass balance of radioactivity to determine if adsorption to the cell culture transport apparatus had occurred. Each sample was placed in 0.9 mL of scintillation fluor (Cytoscint ES, ICN, Cosa Mesa, CA, USA) and read on a Beckman LS 6500 (Palo Alto, CA, USA) scintillation counter for ^3^H activity. For berberine and berbamine analysis, 100 µL aliquot was assayed by HPLC.

For the transport of radiolabeled CsA and digoxin, a 100 µL sample was taken from the receiver chamber over the 3 h period as well as the donor chamber at 3 h to determine the mass balance of radioactivity to assess if adsorption to the cell culture transport apparatus occurred. Each radiolabeled sample was placed in 0.9 mL of scintillation fluor (Cytoscint ES, ICN, Cosa Mesa, CA, USA) and read on a Beckman LS 6500 (Palo Alto, CA, USA) scintillation counter for ^3^H activity.

The apparent permeability (P_app_) was calculated using the following equation [40]:

$$P_{\text{app}}=\text{dQ}/\text{dt}\times 1/\left(A\times C_{0}\right)$$

Where A is the surface area of the monolayer (4.71 cm^2^) and C_0_ is the initial concentration of radiolabeled probe substrate in the donor compartment. dQ/dt is the slope of the steady-state rate constant. The efflux ratio was determined by dividing the P_app_ in the B to A direction by the P_app_ in the A to B direction [41]:

$$\text{Efflux ratio}=P_{\text{app}}\left(B\text{ to }A\right)/P_{\text{app}}\left(A\text{ to }B\right)$$

### Real time quantitative PCR analysis of MDR1 mRNA

Caco-2 or LS-180 cells were grown on Transwell^®^ inserts, 4.71 cm^2^ for 7 (Caco-2) and 6 (LS-180) days. The cells were exposed to E1, E2, levothyroxine or rifampin for 24 h and 48 h. Total RNA was isolated by adding 1 mL Trizol^®^ reagent (Gibco-BRL, Carlsbad, CA, USA) to the cells and processed according to the manufacturer’s instructions. The concentration and purity of isolated RNA samples were measured using Quant-iT^TM^ RiboGreen^®^ RNA assay kit (Invitrogen, Eugene, OR, USA). The RNA sample (0.06 µg) was reverse transcribed by an iScript^TM^ cDNA synthesis kit (Bio-Rad Laboratories, Hercules, CA, USA). Relative quantification of gene expression was performed by iTaq^TM^ SYBR^®^ Green supermix with ROX (Bio-Rad Laboratories) using an ABI Prism 7500 Real Time PCR system (Applied Biosystems, Foster City, CA, USA). Each experiment was performed in duplicate and repeated for each condition tested. The mRNA levels of all genes were normalized using 18s as an internal control. Results are expressed as ratios of MDR1 to 18s expression.

### High performance liquid chromatography (HPLC) analysis

See details of the HPLC analysis method in the Supplementary Material.

### Liquid chromatography/mass spectrometry (LC/MS) analysis

See details of the LC/MS analysis method in the Supplementary Material.

### Statistical analysis

All values are presented as a mean ± standard deviation (SD). The percent of drug transported during the experiment for the various treatments and the P_app_ values within treatment groups were performed with the one-way analysis of variance (ANOVA) followed by a Dunnett’s multiple comparison post-test which compare all the treatments with the control. Differences between A and B transport were compared using a *t*-test. A probability of difference less than 0.05 (*p* < 0.05) was considered to be statistically significant.

## Results

### Cytotoxicity of berberine, berbamine, Oregon grape root extracts

The results of the cytotoxic data indicate that 150 and 300 µM berberine, 300 µM berbamine, significantly decreased MTT reduction and significantly increased LDH leakage in Caco-2 cells. In the MDCKII-MDR1 and MDCKII wild-type cells, none of the berberine and berbamine concentrations were toxic, except for the 300 µM berberine and berbamine in the MDCKII wild-type cells. Thus, concentrations of 100 µM or less of berberine and berbamine were used in these studies.

For Oregon grape root extract 1 (E1) and Oregon grape root extract 2 (E2) only the 2 mg/mL E1 and E2 concentrations were toxic in the Caco-2 cells. Thus, the highest concentration used for the transport studies was 1 mg/mL for E1 and E2. For the real time RT-PCR pretreatment studies in the Caco-2 and LS-180 cells, 0.25 mg/mL or less was used for the 24 and 48 h exposure conditions. See additional details of the results in the Supplementary Material.

### Transepithelial transport of berberine and berbamine

**FIGURE 1:**
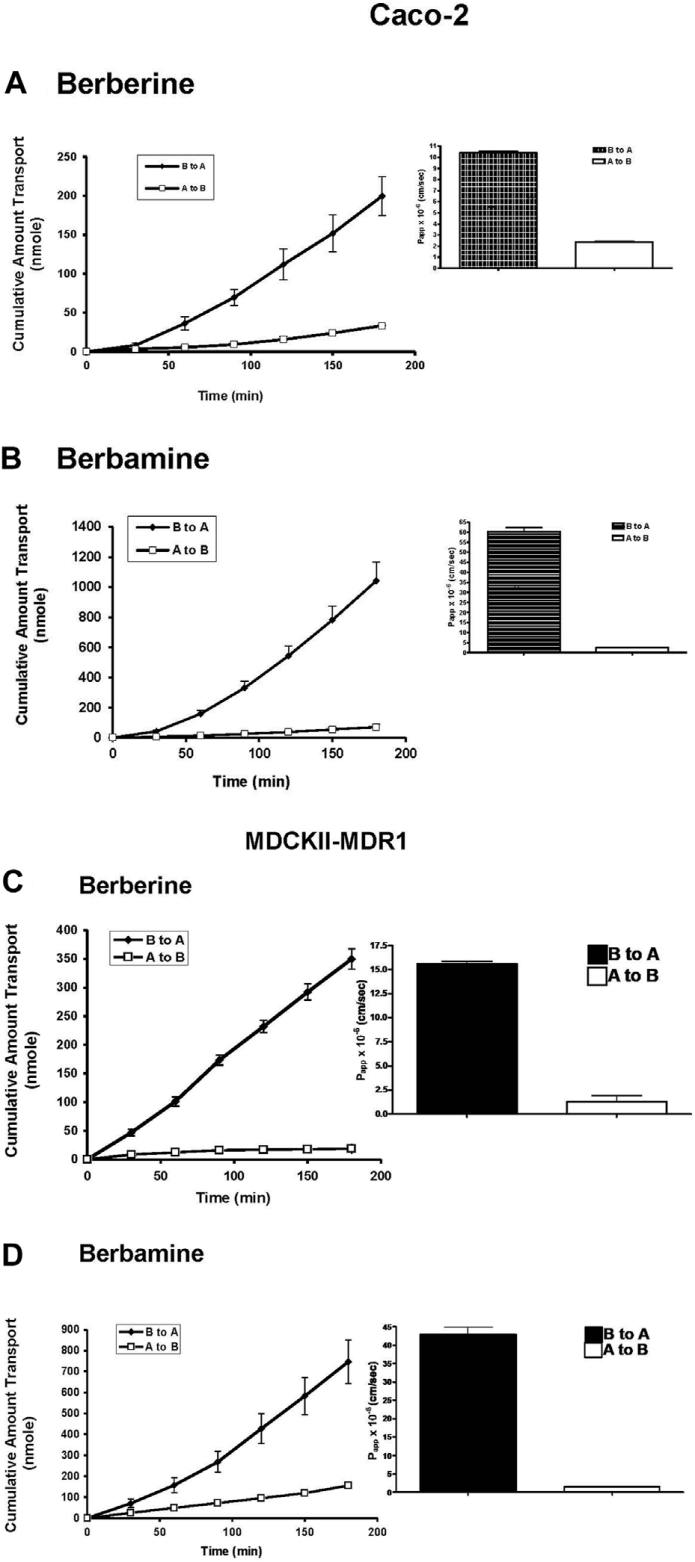
Transport of 100 µM berberine **(A)** or 100 µM berbamine **(B)** from the apical to the basolateral (A to B, □) and the B to A (♦) across Caco-2 cells, and Transport of 100 µM berberine **(C)** or 100 µM berbamine **(D)** from the apical to basolateral (A to B, □) and the B to A (♦) across MDCKII-MDR1 cells. The inset shows the apparent permeability, P_app_, of berberine and berbamine from both transport directions in Caco-2 and MDCKII-MDR cells. Data are represented as mean ± standard deviation, *n* = 4.

In order to investigate whether carrier-mediated transport is involved in the transepithelial transport of berberine and berbamine, transport of 100 µM berberine and berbamine was evaluated as a function of time in the Caco-2 cells and MDCKII-MDR1cells (Figure 1). Because berberine has been shown to be actively effluxed by the Caco-2 cells [19], the efflux mechanism speculated to be involved in berberine and berbamine transport was evaluated by measuring the permeability and the amount transported across the Caco-2 and MDCKII-MDR1 cells from the B to A and the A to B direction. Figures 1A, B show that the transport of berberine and berbamine was faster from the B to A direction (berbine: 1.45 nmol/min; berbamine: 5.94 nmol/min) than that in the A to B direction (berberine: 0.18 nmol/min; berbamine: 0.39 nmol/min) in Caco-2 cells. Similar results were seen with the MDCKII-MDR1 cells (Figures 1C, D). The P_app_ of berberine and berbamine from the B to A direction was also significantly greater than the A to B direction in Caco-2 and MDCKII-MDR1 cells (Figure 1). There was approximately a 4-fold increase of P_app_ observed in the B to A direction (10.4 × 10^−6^ cm/s) compared with the A to B direction (2.35 × 10^−6^ cm/s) for 100 µM berberine (Figure 1A) and ∼30-fold increase of P_app_ in the B to A direction (60.3 × 10^−6^ cm/s) compared with the A to B direction (2.49 × 10^−6^ cm/s) for 100 µM berbamine (Figure 1B). This suggests that there is an efflux mechanism involved in the transport of both berberine and berbamine in the Caco-2 cells. In MDCKII-MDR1 cells, there was a greater efflux seen for berberine than in the Caco-2 cells. An approximately 12-fold increase of P_app_ was observed in the B to A direction (15.6 × 10^−6^ cm/s) compared with the A to B direction (1.3 × 10^−6^ cm/s) (Figure 1C). For berbamine, less efflux was seen in MDCKII-MDR1 cells compared with the Caco-2 cells (Figure 1D).

### Effect of berberine and berbamine on the transport of ^3^H-CsA and ^3^H-digoxin

**FIGURE 2:**
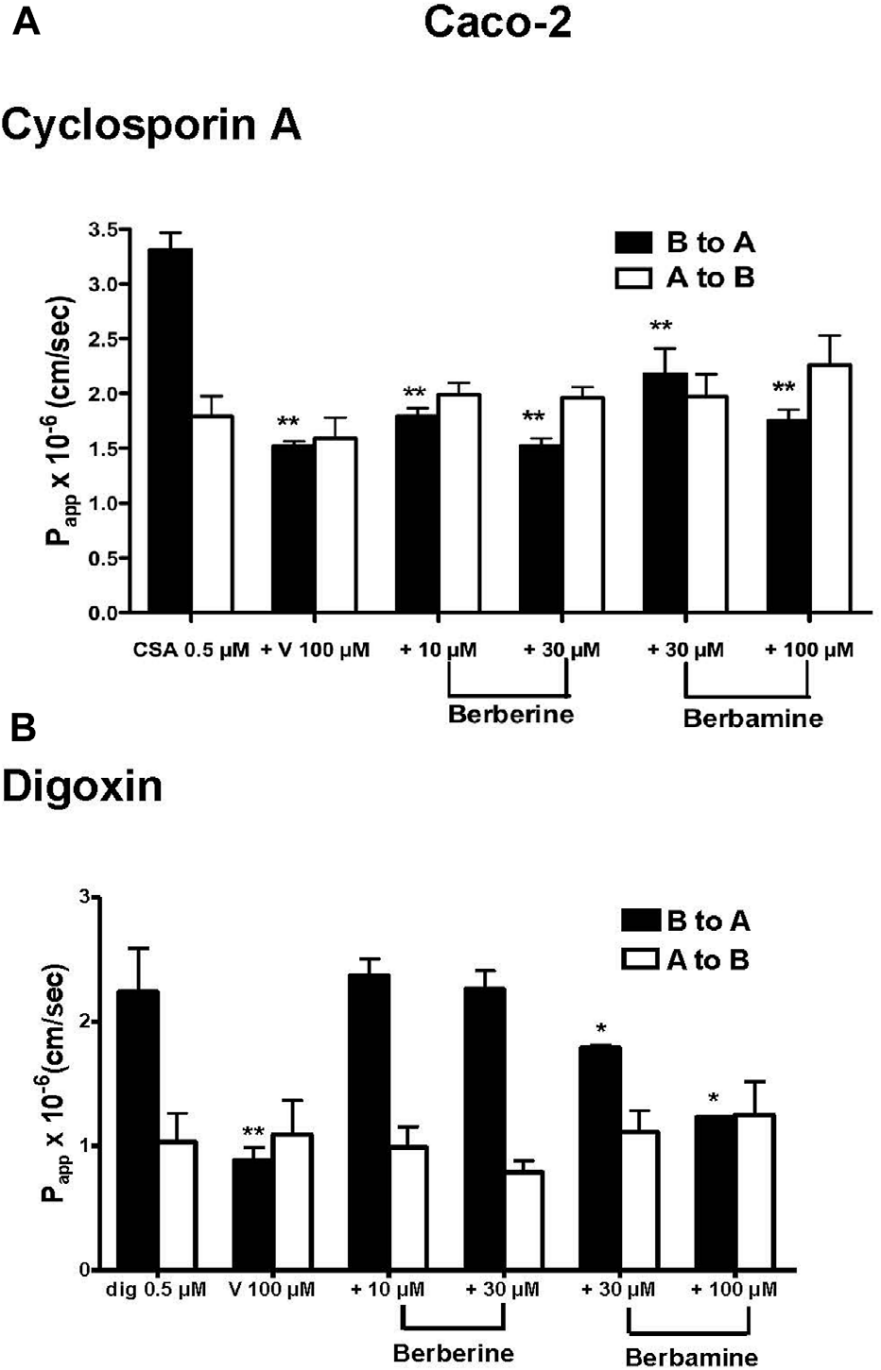
Effects of berberine and berbamine on the transport of 0.5 µM ^3^H-cyclosporin A (CsA, 1 µCi) and 0.5 µM ^3^H-digoxin (dig, 1 µCi) in Caco-2 cells. Panel **(A)** Effects of berberine (10 and 30 µM), berbamine (30 and 100 µM) and 100 µM verapamil (V) on the transport of CsA from basolateral the (B) to apical (A) direction and A to B direction. Panel **(B)** Effects of berberine (10 and 30 µM), berbamine (30 and 100 µM) and 100 µM verapamil on the transport of digoxin from both the B to A and A to B directions. All data represents mean ± standard deviation, *n* = 4. Statistical significance from 0.5 µM CsA or 0.5 µM digoxin is shown with asterisks, *p < 0.05, **p < 0.01.

In the Caco-2 cells, the B to A transport of 0.5 µM ^3^H-CsA was decreased by berberine and berbamine with the significance seen as low as 10 µM for berberine and 30 µM for berbamine (P_app_ B to A of CsA from 2.83 × 10^−6^ cm/s to 2.18 × 10^−6^ cm/s, *p* < 0.0001) (Figure 2A; Table 1). For digoxin, berbamine significantly inhibited efflux of 0.5 µM ^3^H-digoxin (P_app_ B to A of digoxin from 2.78 × 10^−6^ cm/s to 1.79 × 10^−6^ cm/s for 30 µM berbamine, *p* = 0.0253, and to 1.23 × 10^−6^ cm/s for 100 µM berbamine, *p* = 0.0021), whereas there were no significant effects of berberine (up to 30 μM) on digoxin transport (Figure 2B; Table 1). In the MDCKII-MDR1 cells, berberine and berbamine significantly decreased the efflux of 0.5 µM ^3^H-CsA (P_app_ B to A of CsA from 13.10 × 10^−6^ cm/s to 5.34 × 10^−6^ cm/s for 10 µM berberine, *p* < 0.0001, and to 2.55 × 10^−6^ cm/s for 30 µM berbamine, *p* < 0.0001) and the efflux of 0.5 µM ^3^H-digoxin (P_app_ B to A of digoxin from 3.30 × 10^−6^ cm/s to 1.56 × 10^−6^ cm/s for 10 µM berberine, *p* = 0.0232, and to 1.71 × 10^−6^ cm/s for 30 µM berbamine, *p* = 0.0248) (Table 1). Similar results were seen with the MDCKII wild-type cells compared with that in Caco-2 cells (Table 1).

**TABLE 1:** Effect of berberine and berbamine on apparent permeability (P_app_) of ^3^H-cyclosporine A (CsA) and ^3^H-digoxin in Caco-2, MDCKII-MDR1 and MDCKII wild-type (MDCKII-WT) cells from basolateral to apical direction.

Compound	Concentration	Caco-2		MDCKII-MDR1		MDCKII-WT	
	µM	^3^H-CsA	^3^H-digoxin	^3^H-CsA	^3^H-digoxin	^3^H-CsA	^3^H-digoxin
		P_app_ (B to A) (x 10^−6^ cm/s)		P_app_ (B to A) (x 10^−6^ cm/s)		P_app_ (B to A) (x 10^−6^ cm/s)	
Berberine	0	3.31 ± 0.32	2.24 ± 0.70	13.10 ± 1.93	3.30 ± 1.28	6.85 ± 0.88	7.55 ± 0.65
	10	1.79 ± 0.16***	2.37 ± 0.26	5.34 ± 1.56***	1.56 ± 0.43*	4.07 ± 0.56***	7.55 ± 0.46
	30	1.52 ± 0.14***	2.26 ± 0.29	5.17 ± 0.92***	1.57 ± 0.81*	4.34 ± 0.29***	6.95 ± 0.71
	100	1.60 ± 0.45***	2.93 ± 0.07	—	—	—	—
Berbamine	0	2.83 ± 0.28	2.78 ± 0.33	13.10 ± 1.93	3.30 ± 1.28	6.85 ± 0.88	7.55 ± 0.65
	30	2.18 ± 0.46***	1.79 ± 0.04*	2.55 ± 1.14***	1.71 ± 0.66*	4.23 ± 0.54***	4.71 ± 0.80*
	100	1.75 ± 0.21***	1.23 ± 0.02**	2.23 ± 0.95***	1.54 ± 0.72*	4.26 ± 1.01***	4.25 ± 0.30**

Data are represented as mean ± standard deviation. Statistical significance from 0.5 µM ^3^H-CsA and 0.5 µM ^3^H-digoxin shown with asterisks (*p < 0.05, **p < 0.01, ***p < 0.001), *n* = 4. Not determined (—).

### Effect of Oregon grape root extracts on the transport of ^3^H- CsA and ^3^H-digoxin

Table 2 shows the effect of 0.05, 0.1, 0.25, 0.5 and 1 mg/mL E1 or E2 on the transport of 0.5 µM ^3^H-CsA and 0.5 µM ^3^H-digoxin in the Caco-2 cells. The B to A transport of ^3^H- CsA was inhibited by E1 in a dose-response manner with significance effect seen as low as 0.1 mg/mL, *p* = 0.0439, (Table 2). E2 also inhibited the efflux of ^3^H-CsA similarly with significance seen at 0.05 mg/mL (*p* = 0.0007, Table 2). For digoxin, E1 and E2 also inhibited the efflux of ^3^H-digoxin with significance seen at 0.1 mg/mL for E1 (*p* < 0.0001), and 0.05 mg/mL for E2 (*p* < 0.0001). In addition, 0.05 mg/mL E1 significantly increased the absorption of digoxin (*p* = 0.0005). To compare the effect of E1 and E2 modulating the efflux of CsA and digoxin, efflux ratios of the P_app_ of B to A and the A to B transport in Caco-2 cells were determined (Table 2). E1 and E2 significantly decreased the efflux ratios of CsA and digoxin. The effect was seen at 0.1 mg/mL for E1 (*p* = 0.0349) and 0.05 mg/mL for E2 (*p* < 0.0001) for CsA. For digoxin, significance also occurred at 0.1 mg/mL for E1 (*p* = 0.0002) and 0.05 mg/mL for E2 (*p* = 0.0134).

**TABLE 2:** Comparison of apparent permeability (P_app_), and efflux ratio of ^3^H-cyclosporin A (CsA) and ^3^H-digoxin with various concentrations of Oregon grape root extract 1 (E1) and Oregon grape root extract 2 (E2) in Caco-2 cells.

		^3^H-CsA			^3^H-Digoxin		
		P_app_ (B to A) (×10^−6^ cm/s)	P_app_ (A to B) (×10^−6^ cm/s)	Efflux ratio	P_app_ (B to A) (×10^−6^ cm/s)	P_app_ (A to B) (×10^−6^ cm/s)	Efflux ratio
E1	0	5.88 ± 1.98	1.23 ± 0.15	4.67 ± 1.11	5.68 ± 0.30	1.10 ± 0.23	5.31 ± 0.91
	0.05	5.22 ± 1.31	1.47 ± 0.46	3.62 ± 0.45	4.16 ± 0.55	1.95 ± 0.16***	2.13 ± 0.81**
	0.1	4.53 ± 0.72*	1.66 ± 0.60	2.93 ± 0.80*	2.96 ± 0.54***	1.73 ± 0.22**	1.72 ± 0.31***
	0.25	3.15 ± 0.17*	1.61 ± 0.55	2.19 ± 0.96**	2.95 ± 0.57***	1.75 ± 0.26**	1.67 ± 0.08***
	0.5	2.13 ± 0.15*	1.01 ± 0.33	2.27 ± 0.74**	2.98 ± 0.37***	1.84 ± 0.29**	1.67 ± 0.48***
	1	1.68 ± 0.37**	1.65 ± 0.93	1.29 ± 0.71**	2.12 ± 0.26***	2.50 ± 0.25**	0.89 ± 0.09***
E2	0	6.36 ± 0.38	1.18 ± 0.04	5.39 ± 0.20	4.46 ± 0.93	0.59 ± 0.11	7.87 ± 2.45
	0.05	3.99 ± 0.06***	1.22 ± 0.05	3.27 ± 0.12***	2.96 ± 0.83***	0.94 ± 0.14	3.24 ± 1.07*
	0.1	2.28 ± 0.14***	1.61 ± 0.42	1.42 ± 0.43***	2.86 ± 0.99***	0.65 ± 0.18	4.70 ± 2.27*
	0.25	0.83 ± 0.02***	1.28 ± 0.32	0.65 ± 0.22***	2.05 ± 0.84***	0.64 ± 0.22	3.28 ± 0.93**
	0.5	1.09 ± 0.48***	1.30 ± 0.10	0.84 ± 0.43***	2.37 ± 0.95***	0.82 ± 0.10	2.92 ± 1.21**
	1	1.26 ± 0.01***	1.73 ± 0.11	0.72 ± 0.06***	2.02 ± 0.23***	1.26 ± 0.13	1.60 ± 0.19**

Data are represented as mean ± standard deviation. Statistical significance from 0.5 µM ^3^H-CsA and 0.5 µM ^3^H-digoxin shown with asterisks (*p < 0.05, **p < 0.01, ***p < 0.001), *n* = 4.

### HPLC chromatogram and mass spectrometry (MS) of berberine, berbamine and Oregon grape root extracts

It has been reported that Oregon grape root contains berberine. On the other hand, although berbamine was reported to be present in Oregon grape root, it is not a typical component. In order to confirm if it is the case, HPLC was conducted with the Oregon grape root extracts. Berberine was detected in Oregon grape root extracts E1 and E2, but berbamine was not detected in either E1 or E2. See details of the HPLC results in the Supplementary Material.

### Induction of P-gp by Oregon grape root extracts

**FIGURE 3:**
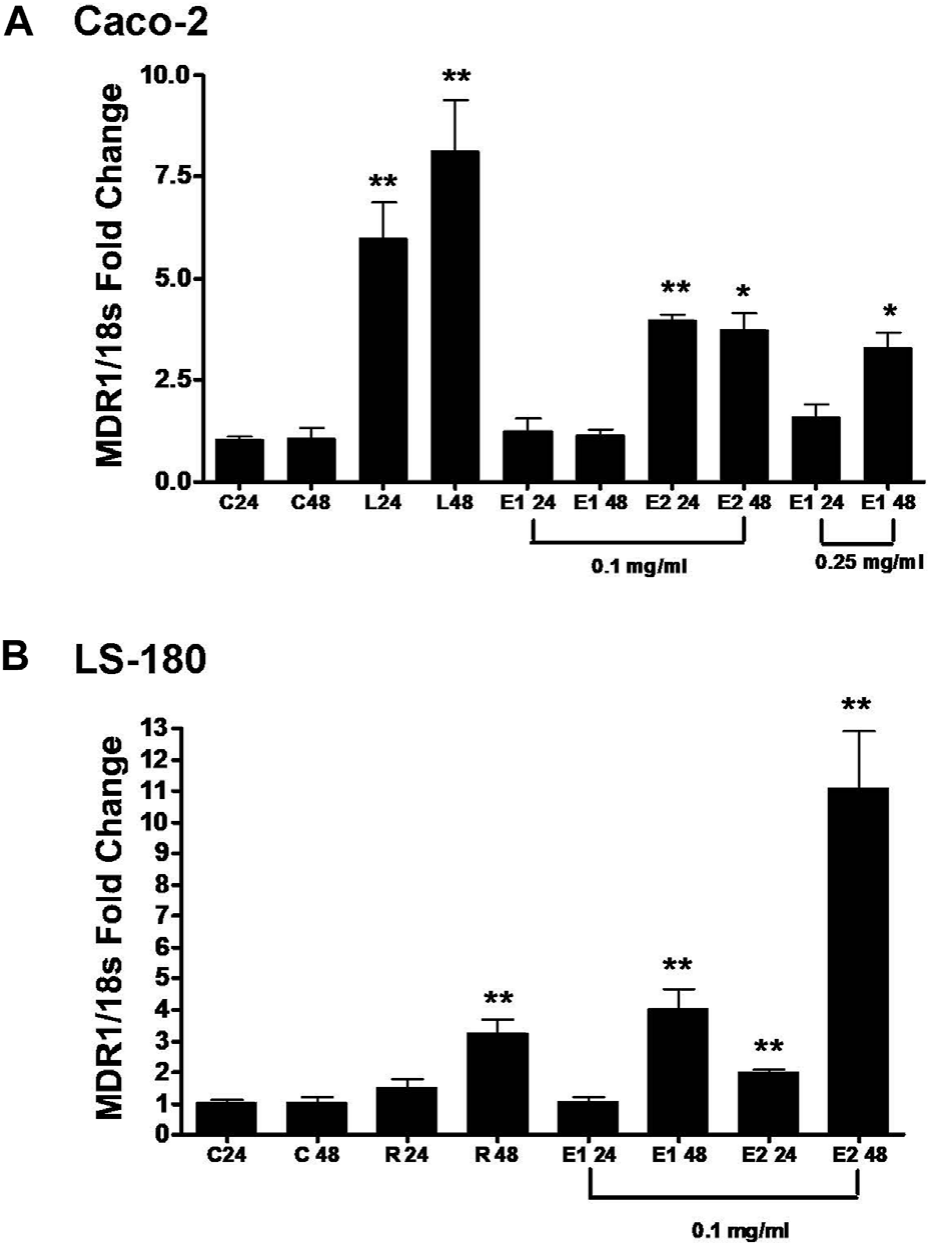
Effect of Oregon grape root extract 1 (E1) and Oregon grape root extract 2 (E2) on the mRNA levels of human MDR1 (ABCB1) in Caco-2 cells (Panel **(A)**) and LS-180 cells (Panel **(B)**). C: control; 24: 24 h; 48: 48 h; L: 100 µM levothyroxine; and R: 10 µM rifampin. Data are represented as mean ± standard deviation, *n* = 4. Statistical significance from control is shown with asterisks (treatments at 24 h were compared with the control at 24 h, treatments at 48 h were compared with control at 48 h), *p < 0.05; **p < 0.01.

In order to investigate the mechanism of induction of P-gp, real time RT-PCR was used in our study. Real time RT-PCR is routinely used to study low abundance gene expression as well as to elucidate the mechanism of modulation of efflux transport proteins [42]. Exposure of Oregon grape root extracts to Caco-2 and LS-180 cells upregulated the mRNA level of the human *MDR1* gene in both cell lines (Figure 3). Levothyroxine, a positive control, upregulated the human *MDR1* gene through a PXR-independent manner in the Caco-2 cells [43]. In Figure 3A, 0.25 mg/mL E1 significantly increased the mRNA level of human *MDR1* at 48 h in the Caco-2 cells (*p* = 0.0312) whereas significance was seen at 0.1 mg/mL E2 at 24 h (*p* < 0.0001). Rifampin was used as the positive control to upregulate the human *MDR1* gene through the PXR-dependent pathway in the LS-180 cells [43]. In Figure 3B, 0.1 mg/mL E1 significantly increased the mRNA level of human *MDR1* in the LS-180 cells at 48 h (*p* = 0.0015) while significance was seen with 0.1 mg/mL E2 at 24 h (*p* = 0.0017) and ∼11-fold increase at 48 h (*p* < 0.0001). This data suggest that E1 and E2 probably upregulate the *MDR1* gene through both the PXR-dependent and PXR-independent pathways.

## Discussion

P-gp, encoded by *MDR1* gene, has been found in both tumor cells as well as epithelial cells of normal tissues which is responsible for the efflux of a large number of structurally and pharmacologically unrelated lipophilic and amphipathic xenobiotics [44], and it also plays a role in drug resistance of cancer chemotherapy [45]. Clinical herb-drug interactions such as St. John’s wort and sertraline have been reported which alter the efficacy or level of adverse effects of the drug [46]. Herbal products have been shown to interact with drug efflux transporters such as P-gp. Examples of interactions are St. John’s Wort [47, 48], garlic [49, 50], ginko biloba extract [51] and milk thistle [52, 53] as P-gp inhibitors or inducers. These herbal products altered the bioavailability of the drugs which are substrates of P-gp, such as CsA and St. John’s Wort [28], saquinavir and garlic [23], and indinavir and milk thistle [54]. It was hypothesized that inhibition of P-gp by herbal constituents may provide a novel approach for reversing multi-drug resistance in tumor cells whereas increased P-gp expression may have therapeutic implications for chemoprotection by herbal medicines.

Bisbenzylisoquinoline alkaloids, which are found in herbal products like goldenseal (*Hydrastis canadensis*), *berberies* and Oregon grape root (*Mahonia aquifolia*), have been shown to interact with P-gp and have the potential for drug-diet interactions [55, 56]. Fu et al. (2001) screened potential MDR modifiers from naturally occurring bisbenzylisoquinoline alkaloids using MCF-7/ADR and KBv200 tumor cells. Many of the natural compounds tested decreased the resistance of tumor cells to doxorubicin and vincristine, P-gp substrates. In addition, these compounds also increased intracellular accumulation of ^3^H-vincristine by 3.3-fold [55]. Moreover, PK11195, an isoquinoline carboxamide ligand, has been shown to increase drug accumulation and facilitate drug-induced apoptosis in human multidrug-resistant leukemia cells [56]. This suggests that the mechanism to reverse MDR is probably linked to increased intracellular drug accumulation by inhibition of P-gp. Also, in adriamycin-resistant mouse leukemia P388/ADR cells and human myelogenous leukemia K562/ADR cells, isoquinoline derivatives effectively reversed the resistance to vinblastine by inhibiting ^3^H-vinblastine efflux by inhibiting the binding of drugs to P-gp [57].

Our study confirms a previous report that berberine is effluxed by the Caco-2 cells and that P_app_ from B to A is greater than A to B direction [18] (Figure 1A). Moreover, the present study shows that berberine is effluxed by the MDCK-MDR1 cells suggesting that berberine is a P-gp substrate, and efflux observed in the Caco-2 is likely mediated by P-gp (Figure 1C). We also found that berberine can inhibit the P-gp-mediated transport of CsA and digoxin (Figure 2). Berberine (10 µM) significantly inhibited the efflux of 0.5 µM CsA in Caco-2 and MDCK-wild type cells with no effect seen on digoxin transport in these two cell systems (Figure 2; Table 1). Similar transport experiments performed with cells overexpressing P-gp, MDCKII-MDR1, demonstrated that berberine significantly inhibits the P_app_ efflux of both CsA and digoxin. The differential effect of berberine observed on the transport of digoxin in the Caco-2 and MDCK-wild type cells vs. MDCKII-MDR1 cells are probably due to multiple mechanisms that are differentially expressed in these cell systems. *In vivo* studies have shown that berberine can alter the bioavailability of P-gp substrates. For instance, the mean area under the curve of CsA was increased 34.5% (*p* < 0.05) after co-administration of 200 mg berberine three times a day for 12 days in renal-transplant patients [58]). In rats, the intestinal absorption of 50 µM berberine was increased with co-exposure of 5 µM CsA or 200 µM verapamil by 12.3% and 14.7%, respectively [59]. *In vitro* studies have shown that berberine can up-regulate P-gp expression in gastric (SC-M1 and NUGC-3) and colon (COLO205 and CT26) cancer cell lines after 24 h exposure [32] as well as increase the intracellular accumulation of rhodamine 123, another P-gp substrate, in bovine brain capillary endothelial cells [33].

Our study provides new evidence that berbamine is effluxed in both Caco-2 and MDCKII-MDR1 cells to a greater extent, ∼5-fold, than berberine (Figures 1B, D). The efflux ratio of berbamine is similar in both the Caco-2 cells (24.2 ± 4.5) and MDCKII-MDR1 cells (27.4 ± 5.1) which indicates that berbamine may be transported by additional efflux transporters in Caco-2 cells. Berbamine was able to significantly inhibit the efflux of CsA and digoxin transport in the Caco-2, MDCKII-MDR1 and MDCKII wild-type cells (Table 1). The effect of berbamine on modulating the transport of CsA and digoxin in the B to A direction was not as potent as berberine in the MDCKII-MDR1 cells.

Oregon grape root extracts are commonly used by Native Americans as a dietary supplement for treating skin disease, gall bladder disease and gastro-intestinal disorder [2, 3, 60]. Currently, there are only two studies of identification of compounds present in the Oregon grape root [61, 62]. One analytical study shows the separation of the Oregon grape root extracts by HPLC but does not quantify any of the components within the extract [61]. Another study used capillary-HPLC coupled with ESI MS/MS for the characterization of alkaloids extracted from Oregon grape root [62]. No publication reports any interactions of Oregon grape root with drugs, metabolic pathways or transporters. Our study provides evidence that berberine was present in both of the Oregon grape root extracts (E1 and E2) and that berberine had similar retention times as the berberine standard with 15% berberine in E1 and 17% berberine in E2. As for berbamine, it was not present in either of the extracts which is inconsistent with a previous study [62]. This could probably be due to the difference in the source of the Oregon grape root extracts and the difference of the analytical method. Further confirmation of these compounds needs to be performed by MS/MS and nuclear magnetic resonance spectroscopy analysis. Unlike berberine, berbamine is not a typical component of Oregon grape root. Our results confirmed that berberine was present in Oregon grape root extracts but not berbamine.

The data from our study supported the hypothesis that Organ grape root extracts would have similar effects as berberine in the modulation of P-gp. Our data show that both E1 and E2 inhibited the efflux transport of CsA and digoxin in Caco-2 cells with E2 being slightly more potent than E1 (Table 2). Because we did not detect berbamine in either E1 or E2, the effect observed may mainly reflect the berberine component in the extracts. The different effect of E1, E2 compared to that of berberine on digoxin transport in Caco-2 cells implies that there may be additional components in E1 or E2 (yet to be determined) that may affect digoxin transport.

We further studied if Oregon grape root extracts can induce P-gp. P-gp expression has been shown to be inducible by many xenobiotics such as rifampin [63], dexamethasone [64], and St. John’s wort [65]. In this study, we used both Caco-2 and LS-180 cells as appropriate *in vitro* models to investigate the mechanism of P-gp induction. Because rifampin can increase P-gp mRNA in LS-180 cells [66], it was used as a positive control in our study. In Caco-2 cells, the positive control, levothyroxine, significantly increase the P-gp mRNA and it is consistent with a previous study [43]. The real time RT-PCR analysis shows that both E1 and E2 up-regulate mRNA levels of human *MDR1* in both Caco-2 and LS-180 cell lines (Figure 3). Because Caco-2 cells lack the PXR receptor while LS-180 cells have it, E1 and E2 probably modulate P-gp through both a PXR-independent and a PXR-dependent manner. Because there are other components in either E1 or E2, whether berberine modulate P-gp in both PXR-independent and PXR-dependent manner needs to be further investigated with the purified berberine component. Based on the recommended dosing regimen for Oregon grape root extracts, 750 mg three times a day, intestinal concentrations of Oregon grape root extracts are approximately 3 mg/mL (dose/250 mL) [65]. Thus, the concentrations evaluated in the study (0.1–1 mg/mL) are clinically relevant assuming berberine levels are similar across extracts. This finding is also consistent with previous findings that berberine induces P-gp. Future clinical studies to understand the potential effect of Oregon grape root extract on the PK of P-gp substrates will help determine the *in vivo* applicability of our *in vitro* findings.

The limitation of our research is that E1 and E2 were from different resources (see Material and Method section), therefore, it is not clear what the exact components are and what different components were between these two extracts. From our analytical results, it is only confirmed that berberine exists in both extracts. Further investigation is warranted to determine all major components and understand the possible differences between the two extracts.

In summary, to the best of our knowledge, this is the first study to show that Oregon grape root extracts can modulate P-gp by inhibiting P-gp-mediated transport and up-regulating mRNA levels of *MDR1,* likely caused by berberine component in the extracts. Thus, dietary/herbal supplements, such as Oregon grape root extracts (that contain berberine), may affect the drug efflux or absorption in the intestine which should be taken into consideration when taking drugs that are substrates for P-gp.

## Figures and Tables

**Table:** 

**ABC**	ATP-binding cassette
**ANOVA**	analysis of variance
**ATCC**	American Type Culture Collection
**BCRP**	breast cancer resistance protein
**BSEP**	bile salt export pump
**CAR**	constitutive androstane receptor
**CsA**	cyclosporin A
**CYP3A4**	cytochrome P450 3A4
**DMEM**	Dulbecco’s Modified Eagle Medium
**E1**	Oregon grape root extract 1
**E2**	Oregon grape root extract 2
**FBS**	fetal bovine serum
**HPLC**	high performance liquid chromatography
**LC/MS**	liquid chromatography/mass spectrometry
**LDH**	L-lactic dehydrogenase
**MATE1**	multidrug and toxin extrusion protein 1
**MDCK**	Madin-Darby Canine Kidney
**MDR**	multi-drug resistance
**MRP**	multi-drug resistance associated protein
**MS**	mass spectrometry
**MTT**	3-(4,5-dimethythiazole-2yl)-2,5-diphenyl tetrazolium bromide
**NTCP**	sodium/taurocholate co-transporting polypeptide (NTCP)
**OATP**	organic anion-transporting polypeptide
**OCT**	organic cation transporter
**P**_**app**_	apparent permeability
**P-gp**	P-glycoprotein
**PXR**	pregnane X receptor
**RT-PCR**	reverse transcriptase polymerase chain reaction
**RXR**	retinoid-X-receptor
**SD**	standard deviation
**TEA**	triethylamine
**TEER**	transepithelial electrical resistance
**β-NADH**	β-nicotinamide adenine dinucleotide, reduced form

## Data Availability

The original contributions presented in the study are included in the article/Supplementary Material, further inquiries can be directed to the corresponding author.

## References

[1] Dattner AM. From medical herbalism to phytotherapy in dermatology: back to the future. Dermatol Ther (2003) 16(2):106–13. 10.1046/j.1529-8019.2003.01618.x 12919112

[2] Bernstein S Donsky H Gulliver W Hamilton D Nobel S Norman R. Treatment of mild to moderate psoriasis with Relieva, a Mahonia aquifolium extract--a double-blind, placebo-controlled study. Am J Ther (2006) 13(2):121–6. 10.1097/00045391-200603000-00007 16645428

[3] Donsky H Clarke D. Relieva, a Mahonia aquifolium extract for the treatment of adult patients with atopic dermatitis. Am J Ther (2007) 14(5):442–6. 10.1097/mjt.0b013e31814002c1 17890932

[4] Chopra BDR Chowan J. Pharmacological action of berberine. Indian J Med Res (1932) 19:1193–203.

[5] Kaneda Y Torii M Tanaka T Aikawa M. *In vitro* effects of berberine sulphate on the growth and structure of Entamoeba histolytica, Giardia lamblia and Trichomonas vaginalis. Ann Trop Med Parasitol (1991) 85(4):417–25. 10.1080/00034983.1991.11812586 1796883

[6] Sanchez-Chapula J. Increase in action potential duration and inhibition of the delayed rectifier outward current Ik by berberine in cat ventricular myocytes. Br J Pharmacol (1996) 117(7):1427–34. 10.1111/j.1476-5381.1996.tb15302.x 8730735 PMC1909453

[7] Iizuka N Miyamoto K Okita K Tangoku A Hayashi H Yosino S Inhibitory effect of Coptidis Rhizoma and berberine on the proliferation of human esophageal cancer cell lines. Cancer Lett (2000) 148(1):19–25. 10.1016/s0304-3835(99)00264-5 10680588

[8] Kuo CL Chi CW Liu TY. The anti-inflammatory potential of berberine *in vitro* and *in vivo* . Cancer Lett (2004) 203(2):127–37. 10.1016/j.canlet.2003.09.002 14732220

[9] Jantova S Cipak L Letasiova S. Berberine induces apoptosis through a mitochondrial/caspase pathway in human promonocytic U937 cells. Toxicol Vitro (2007) 21(1):25–31. 10.1016/j.tiv.2006.07.015 17011159

[10] McDonald MG Tian DD Thummel KE Paine MF Rettie AE. Modulation of major human liver microsomal cytochromes P450 by component alkaloids of goldenseal: time-dependent inhibition and allosteric effects. Drug Metab Dispos (2020) 48(10):1018–27. 10.1124/dmd.120.091041 32591416 PMC7543482

[11] Li Y Li J Yan D Wang Q Jin J Tan B Influence of zuojin pill on the metabolism of venlafaxine *in vitro* and in rats and associated herb-drug interaction. Drug Metab Dispos (2020) 48(10):1044–52. 10.1124/dmd.120.000048 32561594

[12] Li Lhl SY Teh BS Teh B Seow W Thong Y. Anti-inflammatory and immunosuppressive properties of the bis-benzylisoquinolines: *in vitro* comparisons of tetrandrine and berbamine. Int J Immunopharmacology (1989) 11:395–401. 10.1016/0192-0561(89)90086-6 2777433

[13] Sun Y Yao T Li H Peng Y Zheng J. *In vitro* and *in vivo* metabolic activation of berbamine to quinone methide intermediate. J Biochem Mol Toxicol (2017) 31(4):e21876. 10.1002/jbt.21876 27902864

[14] Zhang YT Yu YQ Yan XX Wang WJ Tian XT Wang L Different structures of berberine and five other protoberberine alkaloids that affect P-glycoprotein-mediated efflux capacity. Acta Pharmacol Sin (2019) 40(1):133–42. 10.1038/s41401-018-0183-7 30442987 PMC6318324

[15] Li J Wang Y Hidalgo IJ. Kinetic analysis of human and canine P-glycoprotein-mediated drug transport in MDR1-MDCK cell model: approaches to reduce false-negative substrate classification. J Pharm Sci (2013) 102(9):3436–46. 10.1002/jps.23523 23558561

[16] Zhang H Wang X Wang T Chen K Wang H Jia Q Enhancement of berberine hypoglycemic activity by oligomeric proanthocyanidins. Molecules (2018) 23(12):3318. 10.3390/molecules23123318 30558158 PMC6321252

[17] Zhang X Qiu F Jiang J Gao C Tan Y. Intestinal absorption mechanisms of berberine, palmatine, jateorhizine, and coptisine: involvement of P-glycoprotein. Xenobiotica (2011) 41(4):290–6. 10.3109/00498254.2010.529180 21319959

[18] Maeng HJ Yoo HJ Kim IW Song IS Chung SJ Shim CK. P-glycoprotein-mediated transport of berberine across Caco-2 cell monolayers. J Pharm Sci (2002) 91(12):2614–21. 10.1002/jps.10268 12434406

[19] Nies AT Herrmann E Brom M Keppler D. Vectorial transport of the plant alkaloid berberine by double-transfected cells expressing the human organic cation transporter 1 (OCT1, SLC22A1) and the efflux pump MDR1 P-glycoprotein (ABCB1). Naunyn-Schmiedeberg's Arch Pharmacol (2008) 376(6):449–61. 10.1007/s00210-007-0219-x 18157518

[20] Shi R Yang Y Xu Z Dai Y Zheng M Wang T Renal vectorial transport of berberine mediated by organic cation transporter 2 (OCT2) and multidrug and toxin extrusion proteins 1 (MATE1) in rats. Biopharmaceutics & Drug Disposition (2018) 39(1):47–58. 10.1002/bdd.2112 29065218

[21] Shi R Xu Z Xu X Jin J Zhao Y Wang T Organic cation transporter and multidrug and toxin extrusion 1 co-mediated interaction between metformin and berberine. Eur J Pharm Sci (2019) 127:282–90. 10.1016/j.ejps.2018.11.010 30428337

[22] Sun S Wang K Lei H Li L Tu M Zeng S Inhibition of organic cation transporter 2 and 3 may be involved in the mechanism of the antidepressant-like action of berberine. Prog Neuro-Psychopharmacology Biol Psychiatry (2014) 49:1–6. 10.1016/j.pnpbp.2013.11.005 24246570

[23] Chen C Wu ZT Ma LL Ni X Lin YF Wang L Organic anion-transporting polypeptides contribute to the hepatic uptake of berberine. Xenobiotica (2015) 45(12):1138–46. 10.3109/00498254.2015.1042537 26068524

[24] Wang GFJJ Jin J Zeng J Shi R Dai Y Wu J Involvement of P-glycoprotein and multidrug and toxin extrusion protein 1 in hepatic and renal berberine efflux in mice. RSC Adv (2017) 7(55):34801–9. 10.1039/c7ra01643c

[25] Pedersen JM Matsson P Bergstrom CA Hoogstraate J Noren A LeCluyse EL Early identification of clinically relevant drug interactions with the human bile salt export pump (BSEP/ABCB11). Toxicol Sci (2013) 136(2):328–43. 10.1093/toxsci/kft197 24014644 PMC3858191

[26] Karlgren M Vildhede A Norinder U Wisniewski JR Kimoto E Lai Y Classification of inhibitors of hepatic organic anion transporting polypeptides (OATPs): influence of protein expression on drug-drug interactions. J Med Chem (2012) 55(10):4740–63. 10.1021/jm300212s 22541068 PMC3361267

[27] Nguyen JT Tian DD Tanna RS Hadi DL Bansal S Calamia JC Assessing transporter-mediated natural product-drug interactions via *in vitro*-*in vivo* extrapolation: clinical evaluation with a probe cocktail. Clin Pharmacol Ther (2021) 109(5):1342–52. 10.1002/cpt.2107 33174626 PMC8058163

[28] Tan KW Li Y Paxton JW Birch NP Scheepens A. Identification of novel dietary phytochemicals inhibiting the efflux transporter breast cancer resistance protein (BCRP/ABCG2). Food Chem (2013) 138(4):2267–74. 10.1016/j.foodchem.2012.12.021 23497885

[29] Xiao L Xue Y Zhang C Wang L Lin Y Pan G. The involvement of multidrug and toxin extrusion protein 1 in the distribution and excretion of berberine. Xenobiotica (2018) 48(3):314–23. 10.1080/00498254.2017.1300707 28298174

[30] Kwon M Choi YA Choi MK Song IS. Organic cation transporter-mediated drug-drug interaction potential between berberine and metformin. Arch Pharm Res (2015) 38(5):849–56. 10.1007/s12272-014-0510-6 25359200

[31] Xin HW Wu XC Li Q Yu AR Klotz U. Effects of berberine on the transport of P-gp substrates across Caco-2 and L-MDR1 cell monolayers. Chin Pharmacol Bull (2007)(23) 799–803.

[32] Lin HL Liu TY Lui WY Chi CW. Up-regulation of multidrug resistance transporter expression by berberine in human and murine hepatoma cells. Cancer (1999) 85(9):1937–42. 10.1002/(sici)1097-0142(19990501)85:9<1937::aid-cncr9>3.0.co;2-f 10223233

[33] He L Liu GQ. Effects of various principles from Chinese herbal medicine on rhodamine123 accumulation in brain capillary endothelial cells. Acta Pharmacol Sin (2002) 23(7):591–6. 12100750

[34] Jing W Safarpour Y Zhang T Guo P Chen G Wu X Berberine upregulates P-glycoprotein in human caco-2 cells and in an experimental model of colitis in the rat via activation of nrf2-dependent mechanisms. J Pharmacol Exp Ther (2018) 366(2):332–40. 10.1124/jpet.118.249615 29891588

[35] Han YQYJ Yuan JY Shi YJ Zhu Y Wu SL. Reversal effect of berbamine on multidrug resistance of K562/A02 cells and its mechanism. Zhongguo Shi Yan Xue Ye Xue Za Zhi (2003) 11(11):604–8. 14706144

[36] Tian H Pan QC. A comparative study on effect of two bisbenzylisoquinolines, tetrandrine and berbamine, on reversal of multidrug resistance. Yao Xue Xue Bao (1997) 32(4):245–50. 11499024

[37] Han YQSY Yuan JY Zhu Y Wu SL. The study of reversal resistance effect and its mechanism of berbamine in MCF7/ADR cells. Acta Anatomica Sinica (2004)(35) 161–4.

[38] Evers R Zaman GJ van Deemter L Jansen H Calafat J Oomen LC Basolateral localization and export activity of the human multidrug resistance-associated protein in polarized pig kidney cells. J Clin Invest (1996) 97(5):1211–8. 10.1172/jci118535 8636432 PMC507173

[39] Evers R Kool M van Deemter L Janssen H Calafat J Oomen LC Drug export activity of the human canalicular multispecific organic anion transporter in polarized kidney MDCK cells expressing cMOAT (MRP2) cDNA. J Clin Invest (1998) 101(7):1310–9. 10.1172/jci119886 PMC508708 9525973

[40] Sun J He ZG Cheng G Wang SJ Hao XH Zou MJ. Multidrug resistance P-glycoprotein: crucial significance in drug disposition and interaction. Med Sci Monit (2004) 10(1):RA5–14. 14704647

[41] Crivori P Reinach B Pezzetta D Poggesi I. Computational models for identifying potential P-glycoprotein substrates and inhibitors. Mol Pharmaceutics (2006) 3(1):33–44. 10.1021/mp050071a 16686367

[42] Haslam IS Jones K Coleman T Simmons NL. Induction of P-glycoprotein expression and function in human intestinal epithelial cells (T84). Biochem Pharmacol (2008) 76(7):850–61. 10.1016/j.bcp.2008.07.020 18703021

[43] Mitin T Von Moltke LL Court MH Greenblatt DJ. Levothyroxine up-regulates P-glycoprotein independent of the pregnane X receptor. Drug Metab Dispos (2004) 32(8):779–82. 10.1124/dmd.32.8.779 15258100

[44] Bodo A Bakos E Szeri F Varadi A Sarkadi B. The role of multidrug transporters in drug availability, metabolism and toxicity. Toxicol Lett (2003) 140-141:133–43. 10.1016/s0378-4274(02)00497-6 12676459

[45] Guo A Marinaro W Hu P Sinko PJ. Delineating the contribution of secretory transporters in the efflux of etoposide using Madin-Darby canine kidney (MDCK) cells overexpressing P-glycoprotein (Pgp), multidrug resistance-associated protein (MRP1), and canalicular multispecific organic anion transporter (cMOAT). Drug Metab Dispos (2002) 30(4):457–63. 10.1124/dmd.30.4.457 11901101

[46] Fugh-Berman A Ernst E. Herb-drug interactions: review and assessment of report reliability. Br J Clin Pharmacol (2001) 52(5):587–95. 10.1046/j.0306-5251.2001.01469.x 11736868 PMC2014604

[47] Wang Z Hamman MA Huang SM Lesko LJ Hall SD. Effect of St John's wort on the pharmacokinetics of fexofenadine. Clin Pharmacol Ther (2002) 71(6):414–20. 10.1067/mcp.2002.124080 12087344

[48] Dresser GK Schwarz UI Wilkinson GR Kim RB. Coordinate induction of both cytochrome P4503A and MDR1 by St John's wort in healthy subjects. Clin Pharmacol Ther (2003) 73(1):41–50. 10.1067/mcp.2003.10 12545142

[49] Kaye AD Clarke RC Sabar R Vig S Dhawan KP Hofbauer R Herbal medicines: current trends in anesthesiology practice--a hospital survey. J Clin Anesth (2000) 12(6):468–71. 10.1016/s0952-8180(00)00195-1 11090733

[50] Piscitelli SC Burstein AH Welden N Gallicano KD Falloon J. The effect of garlic supplements on the pharmacokinetics of saquinavir. Clin Infect Dis (2002) 34(2):234–8. 10.1086/324351 11740713

[51] Rosenblatt M Mindel J. Spontaneous hyphema associated with ingestion of Ginkgo biloba extract. N Engl J Med (1997) 336(15):1108. 10.1056/nejm199704103361518 9091822

[52] Gaedeke J Fels LM Bokemeyer C Mengs U Stolte H Lentzen H. Cisplatin nephrotoxicity and protection by silibinin. Nephrol Dial Transplant (1996) 11(1):55–62. 10.1093/oxfordjournals.ndt.a027066 8649653

[53] Schönfeld J Weisbrod B Muller MK. Silibinin, a plant extract with antioxidant and membrane stabilizing properties, protects exocrine pancreas from cyclosporin A toxicity. Cell Mol Life Sci (1997) 53(11-12):917–20. 10.1007/s000180050111 9447243 PMC11147403

[54] Pfaffl MW. A new mathematical model for relative quantification in real-time RT-PCR. Nucleic Acids Res (2001) 29(9):e45–45. 10.1093/nar/29.9.e45 11328886 PMC55695

[55] Fu LW Deng ZA Pan QC Fan W. Screening and discovery of novel MDR modifiers from naturally occurring bisbenzylisoquinoline alkaloids. Anticancer Res (2001) 21(4A):2273–80. 11724282

[56] Jakubikova J Duraj J Hunakova L Chorvath B Sedlak J. PK11195, an isoquinoline carboxamide ligand of the mitochondrial benzodiazepine receptor, increased drug uptake and facilitated drug-induced apoptosis in human multidrug-resistant leukemia cells *in vitro* . Neoplasma (2002) 49(4):231–6. 12382020

[57] Wakusawa S Nakamura S Tajima K Miyamoto K Hagiwara M Hidaka H. Overcoming of vinblastine resistance by isoquinolinesulfonamide compounds in adriamycin-resistant leukemia cells. Mol Pharmacol (1992) 41(6):1034–8. 1614407

[58] Wu LT Tsou MF Ho CC Chuang JY Kuo HM Chung JG. Berberine inhibits arylamine N-acetyltransferase activity and gene expression in *Salmonella typhi* . Curr Microbiol (2005) 51(4):255–61. 10.1007/s00284-005-4569-7 16086103

[59] Pan GY Wang GJ Liu XD Fawcett JP Xie YY. The involvement of P-glycoprotein in berberine absorption. Pharmacol Toxicol (2002) 91(4):193–7. 10.1034/j.1600-0773.2002.t01-1-910403.x 12530470

[60] Moga MM. Alternative treatment of gallbladder disease. Med Hypotheses (2003) 60(1):143–7. 10.1016/s0306-9877(02)00351-1 12450782

[61] Weber HA Zart MK Hodges AE White KD Barnes SM Moody LA Method validation for determination of alkaloid content in goldenseal root powder. J AOAC INTERNATIONAL (2003) 86(3):476–83. 10.1093/jaoac/86.3.476 12852562

[62] Dabrowski D Lech K Jarosz M. Capillary-HPLC with tandem mass spectrometry in analysis of alkaloid dyestuffs - a new approach. Electrophoresis (2018) 39(9-10):1276–83. 10.1002/elps.201700349 29124775

[63] Westphal K Weinbrenner A Zschiesche M Franke G Knoke M Oertel R Induction of P-glycoprotein by rifampin increases intestinal secretion of talinolol in human beings: a new type of drug/drug interaction. Clin Pharmacol Ther (2000) 68(4):345–55. 10.1067/mcp.2000.109797 11061574

[64] Fardel O Lecureur V Guillouzo A. Regulation by dexamethasone of P-glycoprotein expression in cultured rat hepatocytes. FEBS Lett (1993) 327(2):189–93. 10.1016/0014-5793(93)80167-s 8101494

[65] Tian R Koyabu N Morimoto S Shoyama Y Ohtani H Sawada Y. Functional induction and de-induction of P-glycoprotein by St. John's wort and its ingredients in a human colon adenocarcinoma cell line. Drug Metab Dispos (2005) 33(4):547–54. 10.1124/dmd.104.002485 15640377

[66] Schuetz EG Beck WT Schuetz JD. Modulators and substrates of P-glycoprotein and cytochrome P4503A coordinately up-regulate these proteins in human colon carcinoma cells. Mol Pharmacol (1996) 49(2):311–8. 8632764

