# Supplementary material for: Effect of Oregon grape root extracts on P-glycoprotein mediated transport in in vitro cell lines
Source: J Pharm Pharm Sci. 2024 Jan 18;26:11927. doi: 10.3389/jpps.2023.11927 (PMC10830684; doi:10.3389/jpps.2023.11927)
Supplement: Supplementary file 1 [file DataSheet1.docx]

# Supplemental Materials

# Title:

# Effect of Oregon Grape Root Extracts on P-glycoprotein Mediated Transport in In Vitro Cell Lines

# Authors:

YING FAN^1,*, #^, ZHU ZHOU^2,*^, LEI ZHANG^3^

^1^Division of Clinical Review, Office of Safety and Clinical Evaluation, Office of Generic Drugs, Center for Drug Evaluation and Research, U.S. Food and Drug Administration, Silver Spring, MD, USA

^2^York College, The City University of New York, Jamaica, NY, USA

^3^Office of Research and Standards, Office of Generic Drugs, Center for Drug Evaluation and Research, U.S. Food and Drug Administration, Silver Spring, MD, USA

*These authors contributed equally to this work.

Corresponding Author:

^#^Dr. YING FAN,

Telephone: 541-231-5690

Email: fanying12345@gmail.com

**Running title:** Oregon grape root extracts modulate P-glycoprotein

This article reflects the views of the authors and should not be construed to represent FDA’s views and policies. Ying Fan and Lei Zhang are employed by the U.S. Food and Drug Administration. Their contributions to the article were based on their outside research work that does not reflect any position of the U.S. Food and Drug Administration. The content is solely the responsibility of the authors and does not necessarily represent the official views of the National Institutes of Health either.

**Materials and Methods**

*Oregon grape root extract preparation*

Oregon grape root extract 1 (E1) (liquid):

One ml of Oregon grape root extract 1 ethanol solution was transferred to two amber glass vials and 5 ml distilled water was added to each vial and stored at -80˚C for 1 hr. Afterward, the frozen samples were lyophilized using FreeZone® freeze dry system (Kansas City, MO, USA). To prepare E1 stock solution, 100 mg E1 was transferred to a glass vial and 0.25 ml of ethanol (200 proof) and 0.75 ml of distilled water were added and vortexed. Serial dilutions (0.05 - 2 mg/ml) were made with serum-free and antibiotic-free medium for the cytotoxicity and real time RT-PCR studies or with Hanks’ buffered salt solution (HBSS) with 10 mM HEPES and 25 mM D-glucose for the transport studies. The highest percentage of ethanol was 0.15% (v/v). The stock solution was stored at -20˚C. Dilutions were prepared fresh for each experiment and kept away from direct light.

Oregon grape root extract 2 (E2) (root powder):

Exactly 1.5 g of Oregon grape root extract 2 root powder was transferred to an Erlenmeyer flask. Thirty mL methanol was added and sonicated at 25 ˚C for 5 min. The mixture was moderately hand-shaken for 5 min and transferred to two 15 mL centrifuge tubes and centrifuged at 2, 200 rpm for 5 min. The supernatant was collected and stored at 4˚C. To prepare the E2 powder, 1 mL supernatant was transferred to two amber glass vials and 5 mL distilled water was added to each vial. Then, the vials were stored at -80˚C for 1 hr. Afterward, the frozen samples were lyophilized using FreeZone® freeze dry system. Stock solutions were prepared by transferring 100 mg E2 to a glass vial and adding 0.25 mL of ethanol (200 proof) and 0.75 mL of distilled water and then vortexed. Serial dilutions (0.05 - 2 mg/mL) were made with serum-free and antibiotic-free medium for the cytotoxicity and real time RT-PCR studies or with HBSS for the transport studies. The highest percentage of ethanol was 0.15% (v/v). The stock solution was stored at -20˚C. Dilutions were prepared fresh for each experiment and kept away from direct light.

*Chemical exposure*

We initiated cytotoxicity experiments with the Caco-2, MDCKII-MDR1 and MDCKII-wild type cells on Day 7 (Caco-2) and Day 4 (MDCKII-MDR1 and MDCKII-wild type) after the initial plating of the cells at 2.5x10^3^ cells/well in 48-well plates (Becton Dickinson, Franklin Lakes, NJ, USA). Stock solutions of 10 mM berberine or berbamine were prepared by dissolving the compounds in DMSO (berberine) or distilled water (berbamine). Stock solutions of 100 mg/mL E1 or E2 were prepared by dissolving 100 mg E1 or E2 in 25% ethanol. The stock solutions of E1 and E2 were further diluted with serum-free and antibiotic-free medium to yield final concentrations ranging from 0.05 mg/mL to 2 mg/mL for E1 and E2. The final concentration of DMSO was 0.003% (v/v), 0.03% (v/v), 0.3% (v/v), 1% (v/v), 1.5% (v/v), and 3% (v/v) DMSO/culture medium for 0.3 μM, 3 μM, 30 μM, 100 μM, ,150 μM, and 300 μM, respectively. The final concentration of ethanol was 0.15% (v/v) ethanol/culture medium. Ketoconazole (75 µM) was used as a positive control and no treatment (medium alone) was used as a negative control for each experiment.

*Cytotoxicity assays*

Lactate dehydrogenase (LDH) assay and MTT assay

The LDH enzymatic activities were assayed using the method of Mitchell et al.(1). The test samples were obtained from the media after the 4-, 24- and 48-hr E1 and E2 treatments. Enzyme leakage into the medium was expressed as the percentage of total cellular LDH. The MTT (3-(4,5-dimethythiazole-2yl)-2,5-diphenyl tetrazolium bromide) reduction assay was done according to Mosmann (2). This assay was conducted immediately with the same cells used in the LDH study. The results were expressed as the percentage of negative controls.

*High Performance Liquid Chromatography (HPLC) analysis*

A Waters 2690 separations module with a Model 996 photodiode array detector with the Millennium 32 Chromatograph Manager version 3.0 software (Waters Corporation, Milford, MA, USA) was used for HPLC analysis. One mg/mL of E1, E2, berberine and berbamine were prepared by dissolving 1 mg E1, E2, berberine or berbamine in 1 mL methanol:water (50%v/v). All the standards and samples were filtered (0.2 µm) prior to injection. Ten µL was injected onto a 250 × 4.6 mm i.d., 5 µM, Zorbax Eclipse X DB-C_18_ Column (Agilent Technologies, Palo Alto, CA, USA) with a 3.9 × 20 mm i.d. Symmetry® C_18_ guard column, with an isocratic mobile phase of 32:68 acetonitrile:buffer. The buffer consisted of 30 mM ammonium acetate and 14 mM TEA and was adjusted to pH 4.85 with acetic acid. A flow rate of 1 mL/min and UV absorption at 254 nm was used for analysis. The retention time of berberine and berbamine was 7.84 min and 3.03 min, respectively. The cumulative amount of transport was determined using the corresponding peak area for berberine or berbamine. The cumulative amount of berberine or berbamine transported across the cell membrane was evaluated as a functionof time. The P_app_ was calculated by the following equation:

P_app_ = dQ/dt × 1/(A × C_0_)

*Liquid Chromatography/Mass Spectrometry (LC/MS) analysis*

LS/MS analysis was performed using a Surveyor LC pump with Surveyor PDA detector, coupled with Surveyor autosampler system hyphenated to an LCQ advantage mass spectrometer (Thermo Finnigan, San Jose, CA, USA) equipped with an electrospray ionization (ESI) source. The separation was achieved using the same conditions described for the HPLC analysis.

**Results**

*Cytotoxicity of berberine, berbamine, Oregon grape root extracts*

The results of the cytotoxic data are shown in Tables S1 and S2. Table S1 shows the MTT and LDH cytotoxicity data of berberine and berbamine in the Caco-2, MDCKII-MDR1 and MDCKII wild-type cells. After 4 hr exposure to 150 µM and 300 µM berberine, there was a significant decreased reduction of MTT in the Caco-2 cells from 100 ± 0.92% to 76.37 ± 2.13% (*p* < 0.0001) and to 58.25 ± 9.24% (*p* < 0.0001) for 150 µM and 300 µM, respectively. LDH leakage was significantly increased by 150 and 300 µM berberine in the Caco-2 cells (3.80 ± 1.34% to 25.93 ± 4.78%, *p* < 0.0001, and to 44.88 ± 2.09%, *p* < 0.0001, respectively). Three hundred µM berbamine significantly decreased MTT reduction from 100 ± 11.59% to 64.99 ± 11.46% (*p* < 0.0001) and significantly increased LDH leakage from 4.78 ± 0.72% to 43.88 ± 7.44% (*p* < 0.0001) in Caco-2 cells. In the MDCKII-MDR1 and MDCKII wild-type cells, none of the berberine and berbamine concentrations were toxic after 4 hr exposure, except for the 300 µM berberine and berbamine in the MDCKII wild-type cells. Thus, concentrations of 100 µM or less of berberine and berbamine were used in these studies. Lastly, a time-response was also performed at 2, 4, 6, and 8 hr in Caco-2 cells. There was a significant increase in LDH leakage and decrease in MTT reduction as early as 2 hr for 300 µM berbamine (*p* < 0.0001) and 4 hr for 150 µM berberine (*p* < 0.0001) in Caco-2 cells (data not shown).

Table S2 shows dose- and time-response cytotoxicity data of Oregon grape root extract 1 (E1) and Oregon grape root extract 2 (E2) in Caco-2, MDCKII-MDR1 and MDCKII wild-type cells. After 4 hr of exposure, only the 2 mg/mL E1 and E2 concentrations were toxic in the Caco-2 cells. E1 and E2 (2 mg/mL) significantly decreased MTT reduction from 98.23 ± 12.14% to 54.26 ± 2.15% (*p* < 0.0001) and to 32.76 ± 10.76% (*p* < 0.0001), respectively as well as significantly increasing LDH leakage from 2.40 ± 0.49% to 9.99 ± 0.94% (*p* < 0.0001) and to 7.38 ± 1.31% (*p* < 0.0001), respectively. Thus, the highest concentration used for the transport studies was 1 mg/mL for E1 and E2. For the real time RT-PCR pretreatment studies in the Caco-2 and LS-180 cells, 0.25 mg/mL or less were used for the 24 and 48 hr exposure conditions.

*HPLC chromatogram and Mass Spectrometry (MS) of berberine, berbamine and Oregon grape root extracts*

It has been reported that Oregon grape root contains berberine and berbamine. In order to know if it is the case, HPLC was conducted with the Oregon grape root extracts. The HPLC profiles and the total ion chromatograms of berberine and berbamine standards as well as the Oregon grape root extracts, E1 and E2, are shown in Figures S1 and S2, respectively. The retention times and the molecular ion mass ([M+H]) corresponding to berberine and berbamine are indicated in the figures. Peak 2 with a retention time of 7.84 min (Figures S1C and S1D) corresponds to berberine, m/z 336.13 (Figure S2A), was confirmed by co-injection with the standard. Also, MS detection of Peak 2 had the same m/z as the berberine standard in both extracts, E1 and E2 (Figures S2C and S2E). However, berbamine, which has a retention time of 3.03 min, could not be found in either E1 or E2 (Figures S2D and S2F).

**References**

1. Mitchell DB SK, Acosta D. Evaluation of cytotoxicity in cultured cells by enzyme leakage. Journal of Tissue Culture Methods 1980(6):113-6.

2. Mosmann T. Rapid colorimetric assay for cellular growth and survival: application to proliferation and cytotoxicity assays. J Immunol Methods. 1983;65(1-2):55-63.

**Table S1. Berberine and berbamine dose-response cytotoxicity data from the MTT and LDH assays in the Caco-2, MDCKII-MDR1 and MDCKII wild-type (MDCKII-WT) cells after 4 hours exposure****. Values represent the percentage of control.**

**Caco-2 cells MDCKII-MDR1 cells MDCKII-WT cells**

**MTT assay LDH assay MTT assay LDH assay MTT assay LDH assay**

**(%) (%) (%) (%) (%) (%)**

**Berberine**

0.03 µM □ ■ □ ■ □ ■

3.0 µM □ ■ □ ■ □ ■

30 µM □ ■ □ ■ □ ■

100 µM □ ■ □ ■ □ ■

150 µM 76.37 ± 2.13↓ 25.93 ± 4.78↑ □ ■ □ ■

300 µM 58.25 ± 6.37↓ 44.88 ± 2.09↑ □ ■ 24.82 ± 15.91 ↓ ■

**Berbamine**

0.03 µM □ ■ □ ■ □ ■

0.3 µM □ ■ □ ■ □ ■

30 µM □ ■ □ ■ □ ■

100 µM □ ■ □ ■ □ ■

150 µM □ ■ □ ■ □ ■

300 µM 64.99 ± 11.46↓ 43.88 ± 7.44↑ □ ■ 64.32 ± 13.1 ↓ ■

□: %MTT reduction is not significantly decreased compared with the negative control; ■: % Total LDH leakage is not significantly increased compared with the negative control. ↓: %MTT reduction is significantly decreased compared with the negative control (*p* < 0.01); ↑: % Total LDH leakage is significantly increased compared with the negative control (*p* < 0.01). n = 6.

**Table S2. Oregon grape root extract E1 and E2 dose-response cytotoxicity data from the MTT and LDH assays in the Caco-2 and LS-180 cells. Values are expressed as a percentage of control values.**

**Caco-2 Cells** **LS-180 Cells**

**4 hours 24 hours 48 hours 24 hours 48 hours**

**MTT LDH MTT LDH MTT LDH MTT LDH MTT LDH**

**(%) (%) (%) (%) (%) (%) (%) (%) (%) (%)**

**E1**

0.05 mg/ml □ ■ □ ■ □ ■ □ ■ □ ■

0.1 mg/ml □ ■ □ ■ □ ■ □ ■ □ ■

0.25 mg/ml □ ■ □ ■ □ ■ □ ■ □ ■

0.5 mg/ml □ ■ □ ■ □ 12.56↑ □ ■ □ ■

1 mg/ml □ ■ 30.16↓ 11.11↑ 4.73 ↓ 19.28↑ 52.06↓ 27.55↑ 20.35↓ 25.93↑

2 mg/ml 54.26↓ 9.99↑ 1.41↓ 16.54↑ 2.57↓ 19.10↑ 29.46↓ 27.96↑ 11.98↓ 29.49↑

**E2**

0.05 mg/ml □ ■ □ ■ □ ■ □ ■ □ ■

0.1 mg/ml □ ■ □ ■ □ ■ □ ■ □ ■

0.25 mg/ml □ ■ □ ■ □ ■ □ ■ □ ■

0.5 mg/ml □ ■ □ ■ □ 10.21↑ □ ■ □ ■

1 mg/ml □ ■ 16.98↓ 8.32↑ 21.48↓ 12.92↑ 35.24↓ 23.86↑ 33.21↓ 25.85↑

2 mg/ml 32.76↓ 7.38↑ 14.29↓ 9.92↑ 31.70↓ 19.72↑ 19.82↓ 36.13↑ 19.26↓ 40.26↑

□: %MTT reduction is not significantly decreased compared with the negative control; ■: % Total LDH leakage is not significantly increased compared with the negative control. ↓: %MTT reduction is significantly decreased compared with the negative control (*p* < 0.01); ↑: % Total LDH leakage is significantly increased compared with the negative control (*p* < 0.01). n = 5 – 6.
