# Supplementary material for: Effect of Oregon grape root extracts on P-glycoprotein mediated transport in in vitro cell lines
Source: J Pharm Pharm Sci. 2024 Jan 18;26:11927. doi: 10.3389/jpps.2023.11927 (PMC10830684; doi:10.3389/jpps.2023.11927)
Supplement: Supplementary file 2 [file Image1.PDF]

A. Berberine Standard

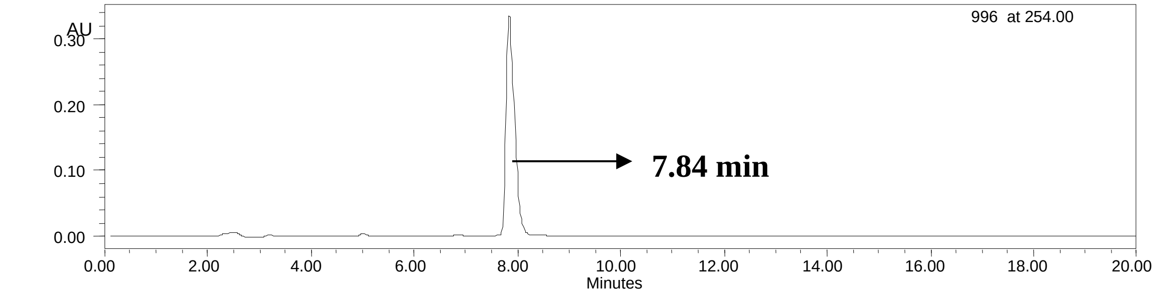

B. Berbamine Standard

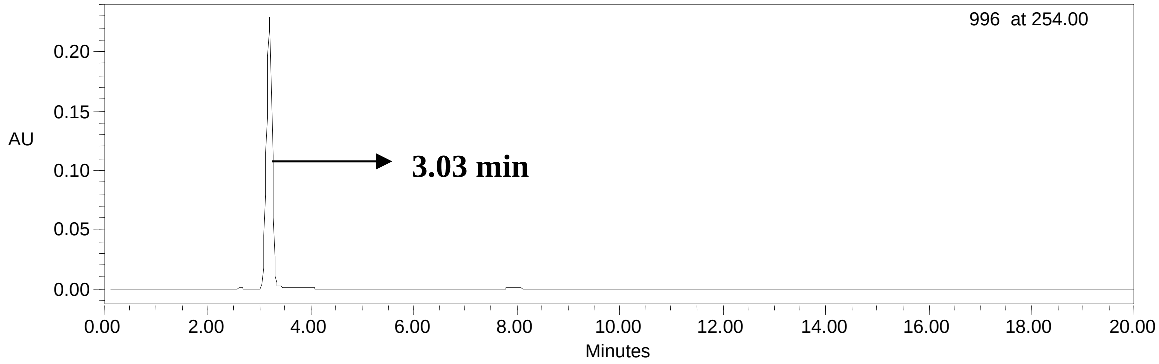

C. Oregon Grape Root Extract 1 (E1)

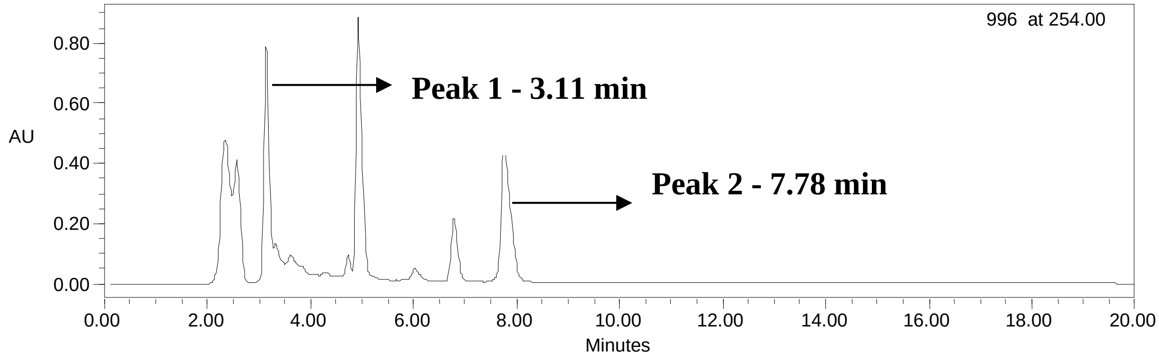

D. Oregon Grape Root Extract 2 (E2)

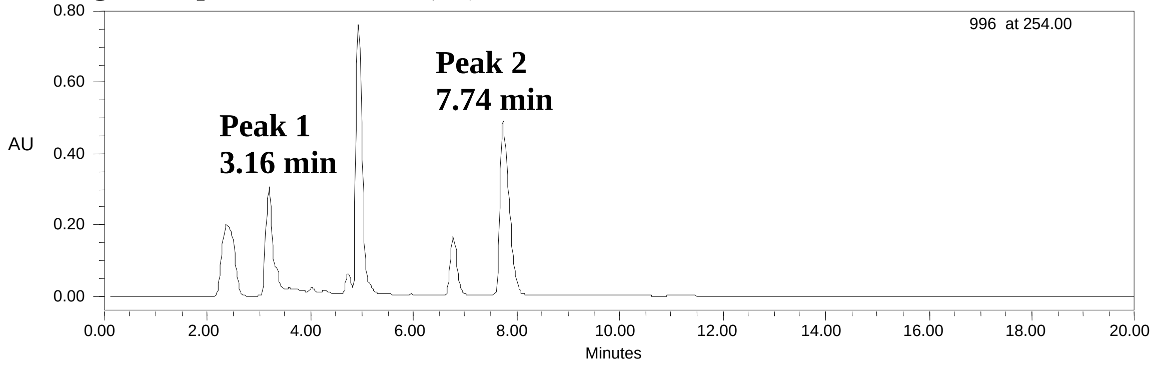

Figure 1S

A. Berberine - retention time 7.84 min

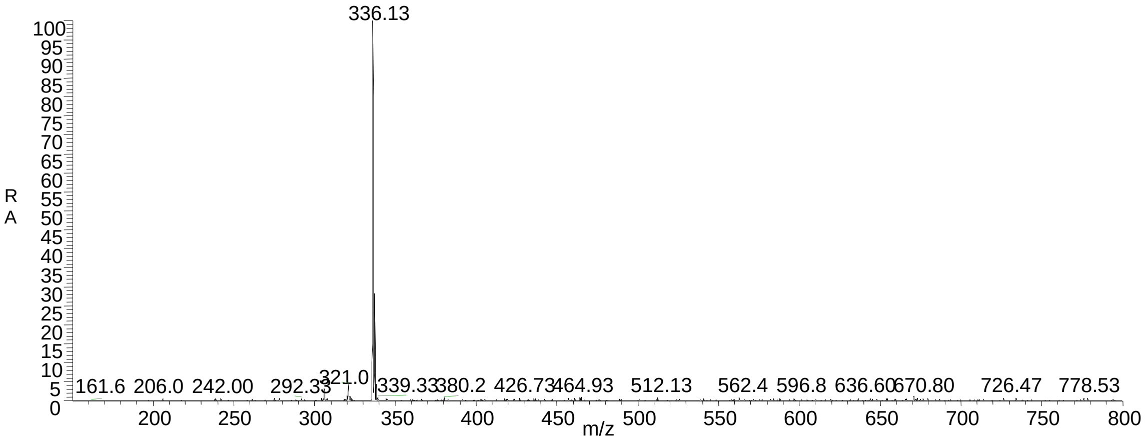

B. Berbamine - retention time 3.03 min

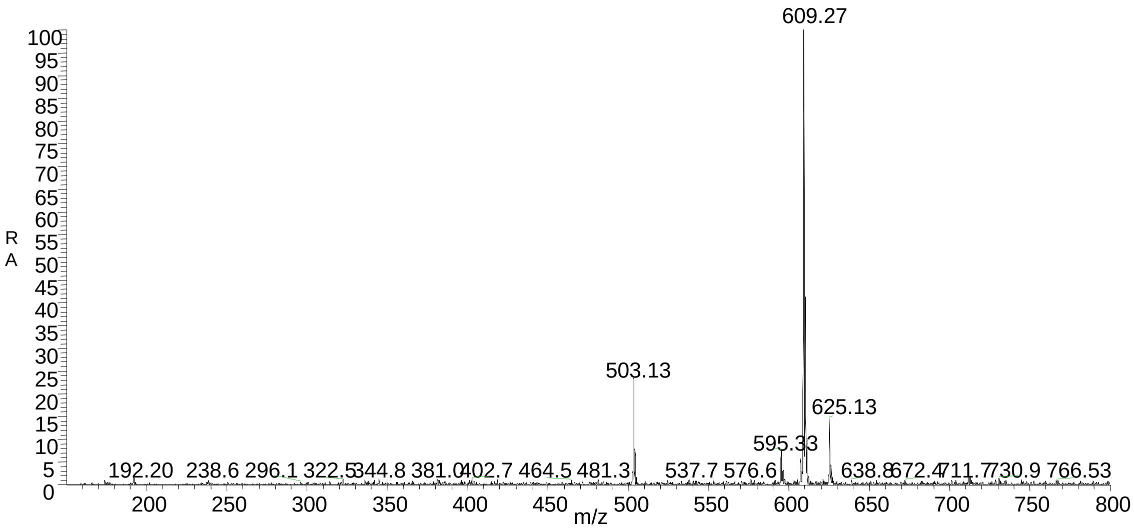

Figure 2S

C. E1 - retention time 7.39 to 8.05 min

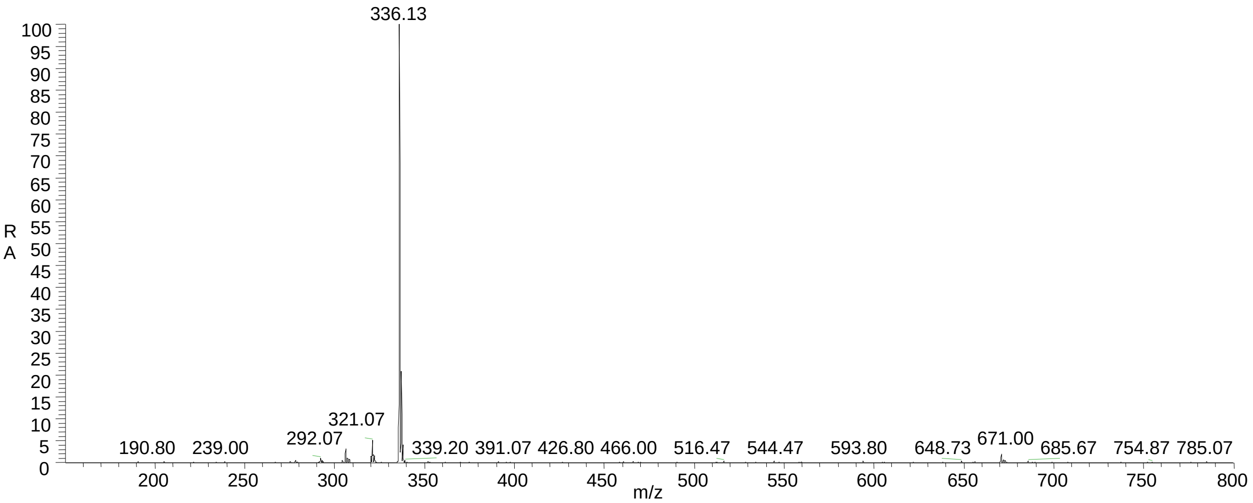

D. E1 - retention time 2.98 to 3.25 min

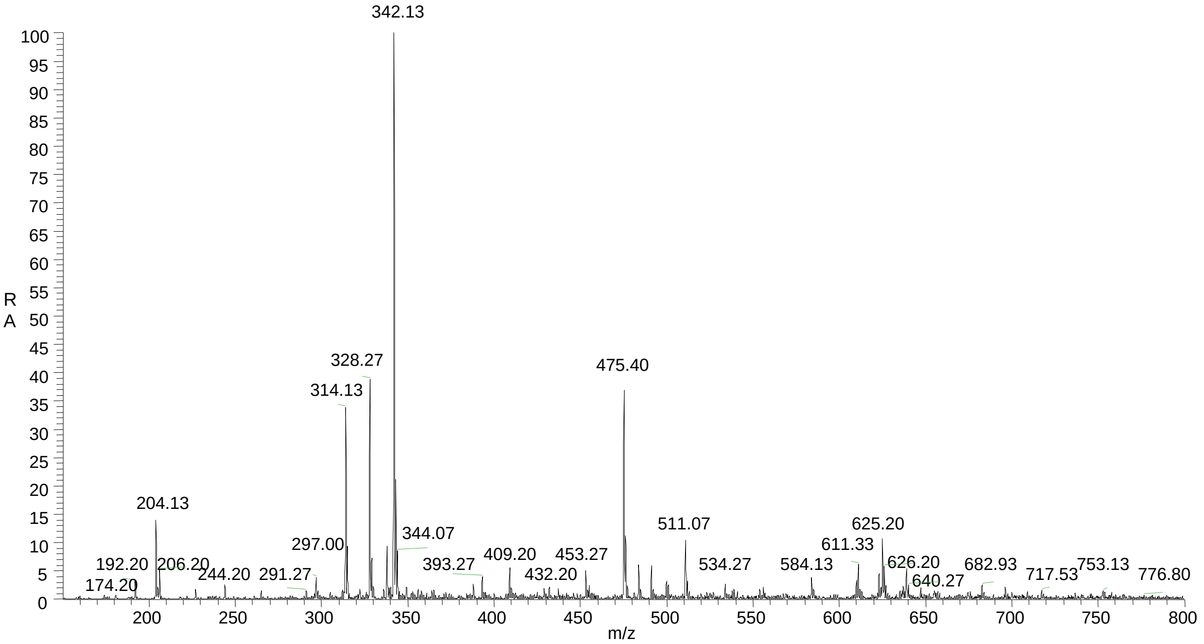

Figure 2S (continued)

E. E2 - retention time 7.12 to 8.36 min

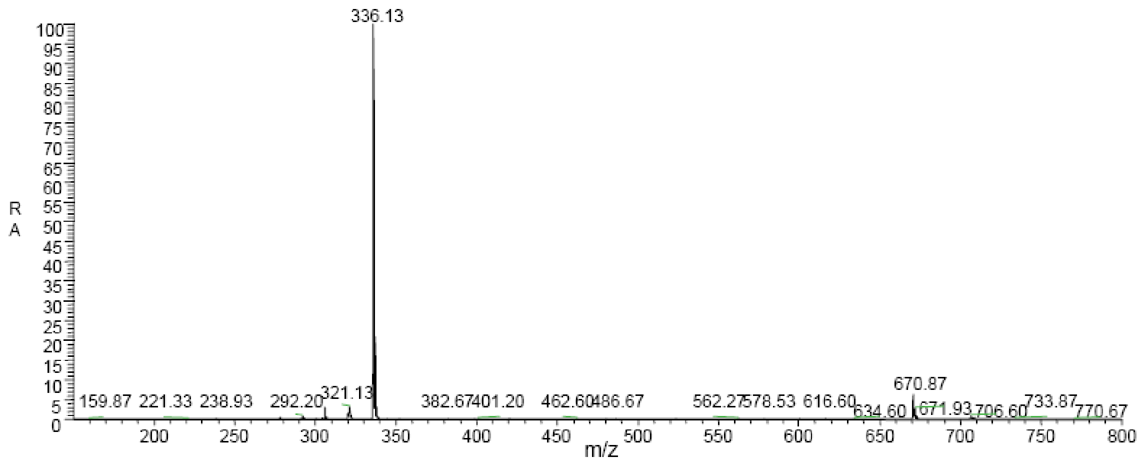

F. E2 - retention time 2.87 to 3.44 min

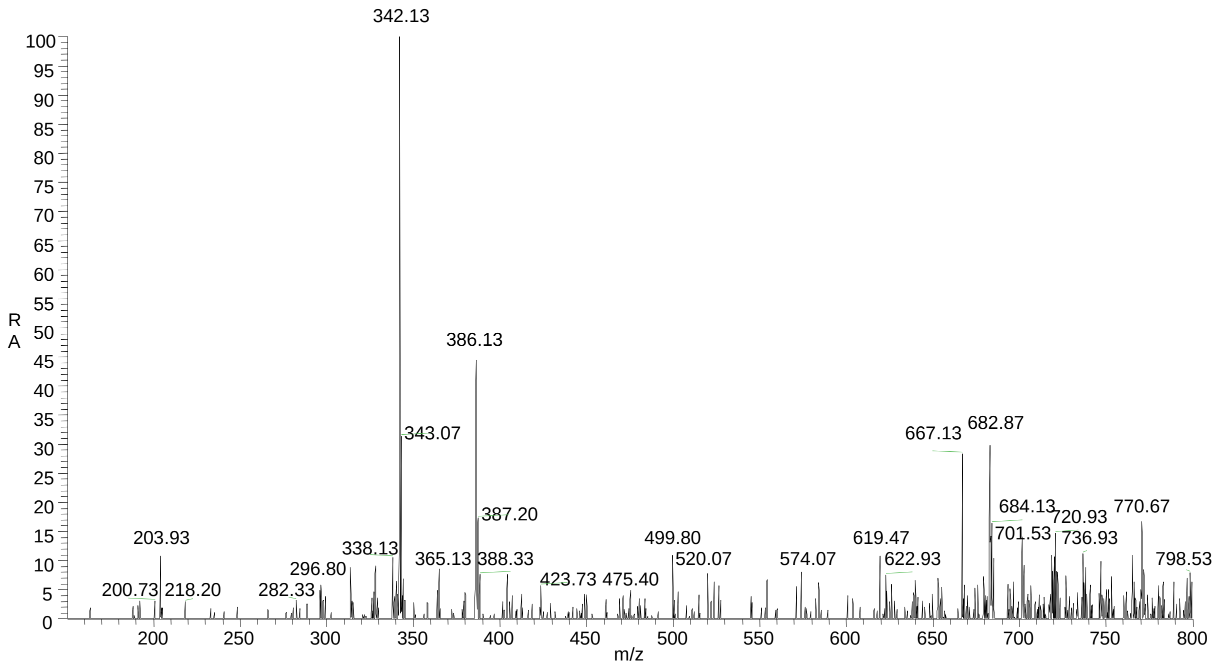

Figure 2S (continued)
